# *Arabidopsis* Spliceosome Factor SmD3 Modulates Immunity to *Pseudomonas syringae* Infection

**DOI:** 10.3389/fpls.2021.765003

**Published:** 2021-12-03

**Authors:** Anna Golisz, Michal Krzyszton, Monika Stepien, Jakub Dolata, Justyna Piotrowska, Zofia Szweykowska-Kulinska, Artur Jarmolowski, Joanna Kufel

**Affiliations:** ^1^Faculty of Biology, Institute of Genetics and Biotechnology, University of Warsaw, Warsaw, Poland; ^2^Department of Gene Expression, Faculty of Biology, Institute of Molecular Biology and Biotechnology, Adam Mickiewicz University, Poznań, Poland

**Keywords:** alternative splicing, biotic stress, miRNA, PAMPS, plant immunity, *Pst* DC3000, RNA-seq

## Abstract

SmD3 is a core component of the small nuclear ribonucleoprotein (snRNP) that is essential for pre-mRNA splicing. The role of *Arabidopsis* SmD3 in plant immunity was assessed by testing sensitivity of *smd3a* and *smd3b* mutants to *Pseudomonas syringae* pv. *tomato* (*Pst*) DC3000 infection and its pathogenesis effectors flagellin (flg22), EF-Tu (elf18) and coronatine (COR). Both *smd3* mutants exhibited enhanced susceptibility to *Pst* accompanied by marked changes in the expression of key pathogenesis markers. mRNA levels of major biotic stress response factors were also altered upon treatment with *Pseudomonas* effectors. Our genome-wide transcriptome analysis of the *smd3b-1* mutant infected with *Pst*, verified by northern and RT-qPCR, showed that lack of SmD3-b protein deregulates defense against *Pst* infection at the transcriptional and posttranscriptional levels including defects in splicing and an altered pattern of alternative splicing. Importantly, we show that SmD3-b dysfunction impairs mainly stomatal immunity as a result of defects in stomatal development. We propose that it is the malfunction of the stomata that is the primary cause of an altered mutant response to the pathogen. Other changes in the *smd3b-1* mutant involved enhanced elf18- and flg22-induced callose deposition, reduction of flg22-triggered production of early ROS and boost of secondary ROS caused by *Pst* infection. Together, our data indicate that SmD3 contributes to the plant immune response possibly *via* regulation of mRNA splicing of key pathogenesis factors.

## Introduction

Plants are challenged by numerous phytopathogens such as bacteria, fungi, and viruses (Muthamilarasan and Prasad, 2013). The best-described hemibiotrophic pathogen is *Pseudomonas syringae* pv. *tomato* DC3000 (*Pst* DC3000), which is the most widely used gram-negative bacterial model to assess plant-pathogen interactions and the principles governing plant resistance (Xin and He, 2013). Highly virulent *Pst* DC3000 usually enters host tissue through wounds or stomatal apparatus in leaves and multiplies rapidly in susceptible plants, like *Arabidopsis thaliana*. Plants prevent the entry of *P. syringae* by stomatal closure, activation of salicylic acid (SA)-dependent basal defense and callose deposition in the cell wall that creates a physical barrier at pathogen infection sites (Melotto et al., 2008; Luna et al., 2011). In turn, *Pst* DC3000 produces phytotoxin coronatine (COR) that activates the jasmonic acid (JA) pathway, induces stomatal reopening and inhibits callose deposition in the cell wall to promote virulence (Melotto et al., 2008; Bari and Jones, 2009; Luna et al., 2011; Zheng et al., 2012; Geng et al., 2014).

In addition to mechanistic barriers, plants have developed a two-step specialized innate immune system. The first step is mediated by extracellular pathogen- or microbe-associated molecular patterns (PAMPs/MAMPs) that activate pattern recognition receptors (PRRs) on the cell surface, resulting in immune responses called pattern-triggered immunity (PTI) (Dodds and Rathjen, 2010; Xin and He, 2013; Macho and Zipfel, 2014; Li et al., 2016; Tang et al., 2017). This response includes PAMP-induced stomatal closure, which is mediated by salicylic acid (SA) and abscisic acid (ABA) signaling (Cao et al., 2011; Lim et al., 2015). The second system, effector-triggered immunity (ETI), is induced by resistance proteins (*R*-proteins), which act as intracellular receptors that recognize avirulence (*Avr*) effectors, often leading to localized programmed cell death (Gimenez-Ibanez and Rathjen, 2010; Li et al., 2016). The best characterized PRR receptor in *Arabidopsis* is a leucine-rich repeat receptor-like kinase (LRR-RLK) FLS2 (*FLAGELLIN SENSITIVE 2*), which recognizes flg22 peptide from bacterial flagellin protein. Flg22 mimics pathogen appearance and causes oxidative stress, callose deposition and ethylene production, leading to the induction of resistance genes [e.g., *PR1* and *PR5* (*PATHOGENESIS-RELATED GENES*), *PAL1* (*PHE AMMONIA LYASE 1*) and *GSTF6* (*GLUTATHIONE S-TRANSFERASE 6*)], but in contrast to the pathogen does not produce the hypersensitive response (HR) type of necrosis (Maleck et al., 2000; Asai et al., 2002; Gomez-Gomez and Boller, 2002). Another well-known PRR is the receptor kinase EFR (ELONGATION FACTOR Tu RECEPTOR), which recognizes an 18 amino-acid (elf18) fragment of the bacterial translation elongation factor EF-Tu. These PRRs initiate immune signaling by heterodimerization with the LRR-RLK family co-receptor BAK1 (BRI1-ASSOCIATED RECEPTOR KINASE) and recruitment of BIK1 (BOTRYTIS-INDUCED KINASE 1) kinase (Chinchilla et al., 2007; Macho and Zipfel, 2014; Couto and Zipfel, 2016; Yeh et al., 2016).

During bacterial infection changes in gene expression result mostly from transcriptional reprogramming, but adaptation to biotic stress occurs also on a post-transcriptional level, including pre-mRNA splicing, mRNA export and degradation (Staiger et al., 2013; Motion et al., 2015; Tsuda and Somssich, 2015; Li et al., 2016; Birkenbihl et al., 2017). A crucial role of splicing in this process is underlined by pathogen response-related phenotypes of mutants defective in alternative splicing (AS) or the contribution of major AS factors, serine/arginine-rich (SR) proteins, and of alternative splicing of some *R*-genes [e.g., *SNC1* (*SUPPRESSOR OF NPR1-1, CONSTITUTIVE 1*) and *RPS4* (*RESISTANCE TO PSEUDOMONAS SYRINGAE 4*)] to pathogen resistance (Zhang and Gassmann, 2007; Zhang X.-N. et al., 2017). Pathogen response can also be modulated by microRNAs (miRNA) that regulate mRNA stability (Ruiz-Ferrer and Voinnet, 2009; Li et al., 2011; Zhang et al., 2011; Weiberg et al., 2014). For example, miR160, miR167, and miR393 are activated by *Pst* infection or flg22 treatment (Navarro et al., 2006), whereas plants lacking or overexpressing miR163 show increased resistance or sensitivity to pathogen, respectively (Chow and Ng, 2017).

SmD3 is one of the core proteins of the spliceosome small nuclear ribonucleoprotein (snRNP) complex. Sm proteins (B/B′, D1, D2, D3, E, F and G) directly bind small nuclear RNAs (snRNAs) and are crucial for splicing initiation by contacting pre-mRNA substrate as a part of U snRNP (Zhang et al., 2001). The *Arabidopsis* SmD3 homologs, SmD3-a and SmD3-b, contain all conserved regions common to SmD3 proteins, including Sm motifs, an RNA binding domain, and C-terminal GRG and RG domains, suggesting that the function of SmD3 in splicing is preserved in *Arabidopsis* (Swaraz et al., 2011). Despite the expression of SmD3-a and SmD3-b in all plant tissues, only the *smd3b* null mutants display pleotropic morphological and developmental phenotypes, including delayed flowering time, reduced root growth and defects in leaf and flower morphology. Consistently, *smd3b* mutation exerts a global effect on pre-mRNA splicing and spliceosome assembly. In contrast, *smd3a* knock-outs have no phenotypic alterations, but the double *smd3a/b* mutant is lethal, suggesting that, although SMD3-b is more important, both proteins have redundant functions.

In this study, we investigated the role of *Arabidopsis* SmD3 protein in response to biotic stress induced by *Pseudomonas syringae* pv. *tomato* DC3000. We show that *smd3a* and *smd3b* mutants are oversensitive to pathogen. Consistently, RNA-seq data for *smd3b-1* plants revealed that lack of SmD3-b protein dysregulates the course of defense against *Pst* DC3000 infection at the level of transcription and splicing of factors involved in different aspects of immune response. Since disease susceptibility of *smd3b-1* plants was observed only after surface inoculation, it appears that mainly pre-invasive stage of defense is attenuated in the mutant, probably resulting from changes in expression of stomatal development and movement genes.

## Materials and Methods

### Plant Material and Growth Conditions

*Arabidopsis thaliana* wild-type ecotype Columbia (Col-0) and *smd3a, smd3b* and *smd1b* mutant plants were used in this study: *smd3b-1* (SALK_006410) was a kind gift of Yoonkang Hur (Chungnam National University, Republic of Korea) (Swaraz et al., 2011); *smd1b* was received from Herve Vaucheret (INRA, CNRS, France) (Elvira-Matelot et al., 2016); *smd3b-2* (SALK_000746), *smd3a-1* (SALK_025193) and *smd3a-2* (SALK_020988) were purchased from NASC. Seeds were surface sterilized and grown on MS medium (Murashige and Skoog, 1962) supplemented with 1% (w/v) sucrose and 0.3% phytogel under 16 h light/8 h dark (long-day) photoperiod at 22/19°C, then seedling were harvested after 2 weeks. Infection experiments were performed on 6-week-old plants grown in soil under an 8 h light/16 h dark (short-day) photoperiod at 22/19°C.

### Bacterial Infection Assays and Pathogen-Associated Molecular Patterns Treatments

Bacterial infection assays were performed with virulent *Pseudomonas syringae* pv. *tomato* strain DC3000 (*Pst*). Bacteria for inoculation were grown overnight in LB medium with rifampicin (50 μg ml^–1^) at 28°C, resuspended in 10 mM MgCl_2_ with density adjusted to 10^6^ cfu ml^–1^ (OD_600*nm*_ = 0.003). 6-week-old plants were inoculated by spraying with *Pst* suspension in MgCl_2_/0.05% Silwet L-77 or with 10 mM MgCl_2_/0.05% Silwet L-77 (control) and covered with plastic lids overnight. Material was harvested from at least 10 plants for each time point, frozen in liquid nitrogen and used for RNA extraction. Bacterial growth was quantified as the number of dividing bacterial cells 24 and 72 h after infection (hpi). Samples (four leaf disks) were taken using a cork-borer (4 mm) from 2 leaves per six plants in each independent replicate.

For PAMP assays sterilized seeds were grown on MS plates as described above. Five days after stratification seedlings were transferred to 24-well plate with liquid MS (two seedlings per well). MS was exchanged for a fresh medium after 8 days and the next day flg22 (Alpha Diagnostic International Inc.), elf18 (synthetized by GL Biochem Ltd., Shanghai, China) or coronatine (Sigma) solution was added to a final concentration of 100 nM. Seedlings were harvested at the indicated time points and frozen in liquid nitrogen.

### RNA Methods

Total RNA was isolated from 2-week-old seedlings or 6-week-old plants using Trizol (Sigma) according to manufacturer’s instructions. Low-molecular weight RNAs were separated on 8% or 15% acrylamide/7 M urea gels and transferred to a Hybond N^+^ membrane (GE Healthcare) by electrotransfer. High-molecular-weight RNAs were analyzed on 1.1% agarose/6% formaldehyde gels and transferred to a Hybond N^+^ membrane by capillary elution. Northern blots were carried out using γ-^32^P 5′-end-labeled oligonucleotide probes or random primed probes prepared using α-ATP^32^ and the DECAprimeTM II labeling kit (ThermoFisher Scientific). Quantification of northern blots was performed using Storm 860 PhosphorImager (GE Healthcare) and ImageQuant software (Molecular Dynamics). Oligonucleotides used for northern hybridization and RT-qPCR are listed in Supplementary Table 2.

### Real-Time PCR

RT-qPCR was carried out on cDNA prepared with mix of Random Primers and oligo-d(T) primer and SuperScript III Reverse Transcriptase (ThermoFisher) from 2 μg of RNA following DNase I digestion (Turbo DNase, ThermoFisher). Quantitative PCR was performed using SYBR Green I Master Mix (Roche) and the LightCycler 480 (Roche). Results were normalized to *UBC9* (*At4g27960*) or *GAPDH* (*At1g13440*) mRNAs.

### RNA-seq

Samples for RNA-seq were collected 48 h after infection and total RNA was isolated using Trizol as described above. RNA quality was verified with the Bioanalyzer 2100 (Agilent). Libraries were prepared from three independent biological replicates using Illumina TruSeq Stranded Total RNA with Ribo-Zero Plant rRNA Removal (Plant Leaf) protocol including barcoding and were paired-end sequenced by OpenExome s.c. (Poland). The quality of the data was assessed using *fastqc* (v0.11.2^1^). Reads for each sample were aligned to the TAIR10 *A. thaliana* genome from ensemble (release v29; Kersey et al., 2016) and reference annotation AtRTD2 (release 19.04.2016; Zhang R. et al., 2017) using STAR (v2.5.0a, Dobin et al., 2013) with the following command-line parameters:

*STAR - -runMode alignReads - -sjdbOverhang 149 - -sjdbGTF file - -readFilesCommand - -sjdbInsertSave All - -outFilterType BySJout - -outFilterMultimapNmax 10 - -alignSJoverhangMin 10 –outFilterMismatchNmax 10 - -alignIntronMax 100000 - -align MatesGapMax 100000 - -outSAMattrIHstart 0 - -outMultimapper Order Random* - -outSJfilterIntronMaxVsReadN 5000 *10000 15000 20000 - -outSAMtype BAM SortedByCoordinate - -quant Mode GeneCounts - -outSAMunmapped Within - -sjdbFileChr StartEnd.*

RNA-seq alignments were split to separate read-pairs that originate from transcription on the forward and reverse strands using samtools (v1.1; Li et al., 2009). Coverage graphs were calculated with *genomeCoverageBed* from *bedtools* (v2.17.0; Quinlan and Hall, 2010) with normalization to number of reads and were converted to bigwig format with bedGraphToBigWig (v4^2^). Differential expression (DE) was performed using *DESeq2* (v1.16.1) *R* (v3.4.1) package with parameter *alpha* = *0.05* (Love et al., 2014). Genes with FDR adjusted *p*-value < 0.05 and absolute log_2_FC > 1 were considered significantly changed. Clustering of gene expression profiles was performed using standard R functions on sets of genes selected based on their expression change and GO term affiliation. Splicing analysis was done using reference annotation AtRTD2 (release 19.04.2016; Zhang R. et al., 2017) and rMATS (v3.2.5; Shen et al., 2014) with command-line parameters: *-t paired -len 149 -libType fr-firststrand -novelSS 1.* Differential splicing events with FDR < 0.05 and delta PSI > 0.05 were considered as significant. Sashimi plots were created using IGV from RNA-seq data (Thorvaldsdóttir et al., 2013). RNA-seq data reported in this article have been deposited in the Gene Expression Omnibus database under Accession Number GSE117077.

### Callose Deposition Assay

Approximately ten sterilized Col-0 and *smd3b* seeds were sown per well in 6-well plates, containing MS medium and grown under long-day conditions for 7 days, when the medium was replaced by fresh MS. Plants were treated with flg22, elf18, and coronatine as described (Luna et al., 2011) at the final concentration of 1 μM for 24 h. Samples were washed with 95% EtOH and incubated for 2 h in 0.07 M K_2_HPO_4_ containing 0.01% aniline-blue (Sigma). Imaging was performed using a fluorescence microscope with DAPI filter at a wavelength of 370 nm and images were analyzed using the ImageJ software.

### Measurement of ROS Production

ROS production was detected using GloMax^®^-Multi^+^ Detection System (Promega) according to published protocols with minor modifications (Smith and Heese, 2014; Bisceglia et al., 2015). Leaf disks cut from 6-week-old plants were floated in water in individual wells of a 96-well microplate for 24 h to reduce wounding response. Immediately prior to elicitation, water was replaced with luminol/peroxidase/*Pst* or only luminol/peroxidase when injector was charged with the 5x flg22 solution. The luminescence detection assays were performed for at least 30 min with 1 sec signal integration time.

## Results

### Lack of *SmD3* Affects Resistance to *Pst* DC3000 Infection

**FIGURE 1:**
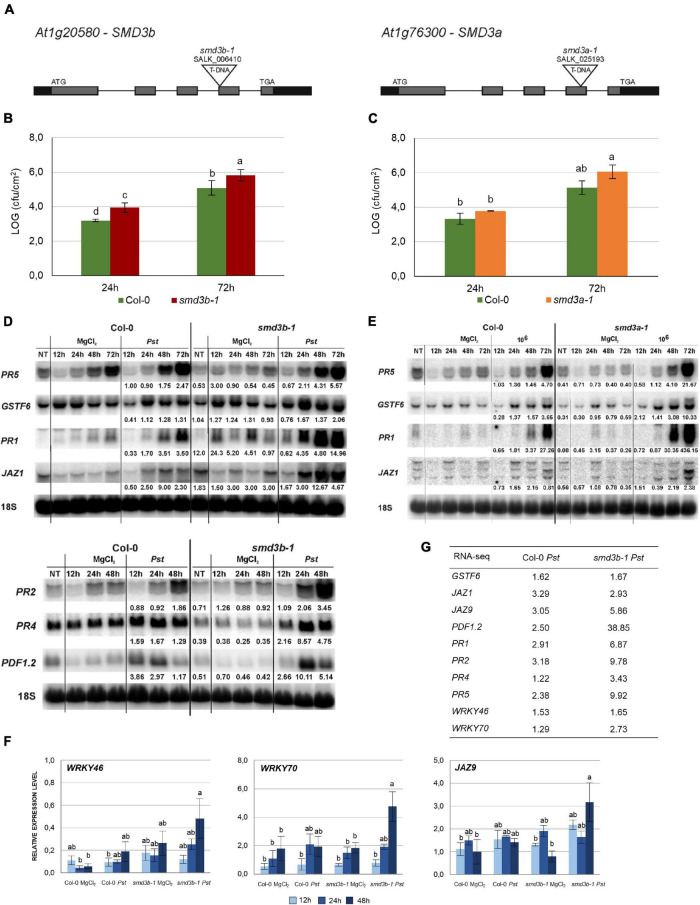
The *smd3b-1* and *smd3a-1* mutants are susceptible to *Pseudomonas syringae* pv. *tomato* DC3000 infection. **(A)** Structure of the *AtSMD3-B* (*At1g20580*) and *AtSMD3-A* (*At1g76300*) genes. Exons are represented by gray bars, UTRs by black bars and localization of T-DNA insertions are indicated. **(B,C)** Growth of *Pst* DC3000 after 24 and 72 hpi in Col-0 and the *smd3b-1* **(B)** or *smd3a-1* **(C)** mutant. For each time point leaf disks were collected from 5 plants. Results are mean of four independent experiments and error bars represent SEM; letters represent significant difference (*P* < 0.05) for Tukey’s HSD test. **(D,E)** Northern blot analysis of factors involved in response to *Pst* DC3000. Samples were collected from non-treated (NT), control (MgCl_2_) and infected (*Pst*) Col-0 and *smd3b-1* **(D)** or *smd3a-1* **(E)** plants at indicated time points. Numbers represent the ratio of transcript level in *Pst-*treated Col-0 and mutants relative to control and normalized to 18S rRNA loading control. Experiments were repeated at least three times; representative blots are shown. **(F)** RT-qPCR analysis of selected pathogen response genes in *smd3b-1*. Mean values ± SEM were obtained from three independent experiments; letters represent significant difference (*P* < 0.05) for Tukey’s HSD test. *UBC9* mRNA was used as a reference. **(G)** The expression levels of selected pathogen response genes in Col-0 and the *smd3b-1* mutant based on RNA-seq analysis. Numbers represent fold change.

To investigate the function of SmD3-b protein in plant innate immunity we tested the resistance of the *Arabidopsis smd3b* T-DNA insertion mutants, *smd3b-1* (SALK_006410, Figures 1A,B) and *smd3b-2* (SALK_000746, Supplementary Figures 1A,B) to *Pst* DC3000 infection by spraying. Upon infection, chlorotic and necrotic symptoms were visible at 72 hpi (hours post infection) both in the wild-type (Col-0) and the *smd3b-1* mutant, but were more severe in the mutant (Supplementary Figure 1C). Bacteria growth assayed after 24 and 72 hpi showed that both *smd3b-1* and *smd3b-2* mutations resulted in an increased pathogen multiplication compared to the wild-type (Figure 1B and Supplementary Figure 1B). As the effect was slightly weaker in the case of the *smd3b-2* line, we used the *smd3b-1* mutant for further analyses. Although *smd3a* knock-out has no phenotypic consequences (Swaraz et al., 2011), we also checked the effect of *Pst* on *smd3a-1* (SALK_025193, Figures 1A,C) and *smd3a-2* (SALK_020988, Supplementary Figures 1A,B) mutants. Both *smd3a* lines exhibited increased sensitivity to *Pst* compared to the wild-type, but these changes were not statistically significant (Figure 1C and Supplementary Figure 1B). These results indicate that plants lacking SmD3 are more susceptible to hemibiotrophic bacterial infection. To assess cellular defense to the pathogen in *smd3b* and *smd3a* plants we investigated changes in mRNA levels of key pathogenesis markers: *PR1*, *PR2*, *PR4*, *PR5, PDF1.2* (*PLANT DEFENSIN 1.2*) and *GSTF6* that are involved in the SA response, and two *JASMONATE-ZIM-DOMAIN PROTEINS JAZ1* and *JAZ9* from the JA pathway, which are induced by coronatine in a specific mechanism used by the *Pst* DC3000 strain to manipulate jasmonate signaling (Lieberherr et al., 2003; Demianski et al., 2011; Barah et al., 2013). Northern blot and RT-qPCR confirmed that these markers were activated after infection in both Col-0 and the mutants, however, this effect was stronger in *smd3b* and *smd3a* plants after 48 and 72 hpi compared to the wild-type (Figures 1D–F and Supplementary Figures 1D,E, 2). Also, accumulation of three major WRKY transcription factors mRNAs (*WRKY46*, *WRKY53*, and *WRKY70*), that are involved in defense response *via* the SA pathway and modulate systemic acquired resistance (SAR) (Wang et al., 2006), was more prominent in the *smd3b-1* mutant following infection (Figure 1F and Supplementary Figure 1E). In contrast, expression of other pathogen response-related factors, *SGT1* (*SALICYLIC ACID GLUCOSYLTRANSFERASE 1*), *NPR1* (*NON-EXPRESSER OF PR GENES 1*), *NPR3* (*NPR1-LIKE PROTEIN 3*) was not significantly altered in the *smd3b-1* mutant (Supplementary Figure 1E). Together, these results provide evidence that lack of SmD3 protein dysregulates the response to *Pst* DC3000 infection.

Since SmD3-b is a core component of the snRNP complex, we wondered whether other Sm proteins have a similar impact on plant innate immunity. We therefore tested susceptibility to *Pst* infection of the *smd1b* mutant in another Sm protein, SmD1-b. Bacteria growth assay showed that *smd1b* plants were significantly more sensitive to *Pst* compared to the wild-type, with a similar level of pathogen proliferation as observed for *smd3b* and *smd3a* (Figures 1B,C and Supplementary Figures 1B, 3A). Moreover, as was the case for *smd3b* and *smd3a* lines, activation of key pathogenesis markers, *PR1*, *PR5*, *GSTF6* and *JAZ1*, was also stronger in *smd1b* plants than in Col-0 (Supplementary Figure 3B). These results reinforce the notion that Sm core splicing factors, most likely as a spliceosomal complex, contribute to shaping the scope of pathogen response signaling.

### The Impact of *SmD3-b* on Gene Expression in Response to *Pst* DC3000 Infection

**FIGURE 2:**
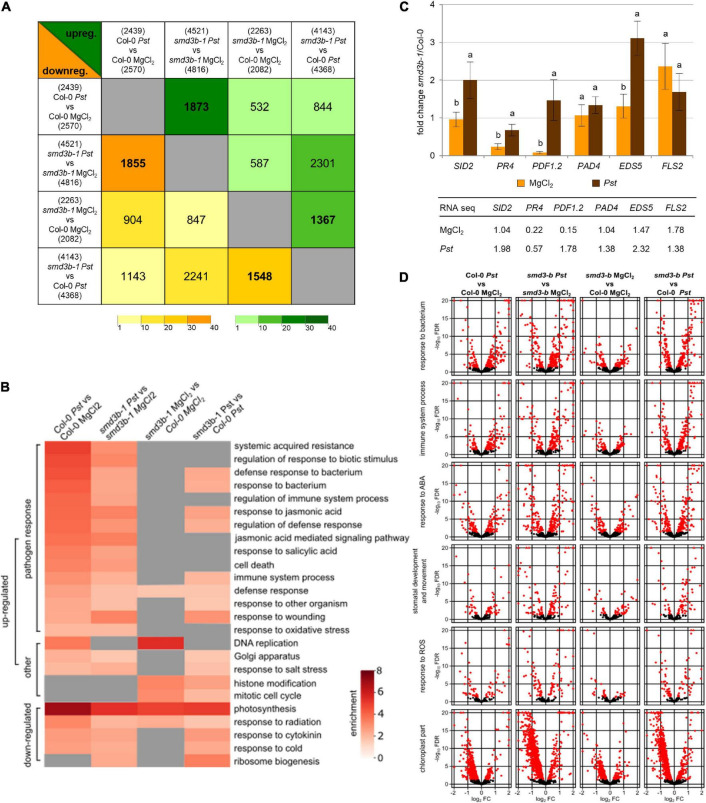
Genes affected by *Pst*-infection and lack of SmD3-b. **(A)** Affected genes show high overlap between different comparisons. Comparison of genes with changed expression in RNA-seq data for control (MgCl_2_) and infected (48 hpi; *Pst*) Col-0 and *smd3b-1* plants using odds ratio statistical test, which represents the strength of association between the two lists (odds ratio ≤ 1, no association; odds ratio > > 1, strong association). Number of genes with changed expression is shown above (upregulated) and below (downregulated) headings of each comparison; number of overlapping genes is shown in each cell for upregulated (green triangle) and downregulated (yellow triangle) genes, respectively. Color gradients mark for odds ratio calculated with GeneOverlap R package. **(B)** Genes with changed expression (FDR < 0.05) show enrichment in several GO categories (shown are only chosen categories, all results are in Supplementary Dataset 2). **(C)** Comparison of expression changes for selected mRNAs by RT-qPCR and RNA-seq. Plot show fold changes of transcript levels in *smd3b-1* vs. Col-0 in control condition (MgCl_2_) and 48 hpi (*Pst*). RT-qPCR values represent mean of three independent biological replicates with error bars showing standard deviations (*SD*); letters represent significant difference (*P* < 0.05) for Tukey’s HSD test. *UBC9* mRNA was used as a reference. Table presents analogous results from RNA-seq. **(D)** Bacterial infection and the *smd3b-1* mutation impact gene expression in selected categories. Volcano plots (-log_10_FDR as a function of log_2_FC) for genes implicated in response to bacterium, immune system process, response to abscisic acid (ABA), stomatal development and movement, response to ROS and chloroplast part. Red dots and triangles mark genes with significant changes.

To estimate the impact of *smd3b-1* mutation on the cell transcriptome under normal conditions and during infection, we sequenced RNA samples from 6-week-old mutant and Col-0 control plants and plants treated with *Pst* DC3000. Analysis of differential gene expression revealed a significant number of affected genes (DESeq2, FDR (false discovery rate) < 0.05; Supplementary Figure 4A and Supplementary Dataset 1) between wild-type and the mutant and among treatments. These results show that both *Pst* infection and lack of SmD3-b protein have profound effects on *Arabidopsis* gene expression. RNA-seq data also confirmed changes in mRNA levels as measured by northern blot and RT-qPCR, except for *JAZ1* that was downregulated in RNA-seq (see Figure 1G). Principal component analysis (PCA) confirmed that the results of sequencing created four coherent groups of biological replicas (Supplementary Figure 4B). Sets of affected genes show strong overlaps when compared using an odds ratio statistical test (Figure 2A; all odds ratio > 2.5; GeneOverlap R package Shen and Sinai, 2013). Interestingly, similarity of the lists of genes with changed expression was not limited to sets representing response to infection or impact of *smd3b-1* mutation, but there was also some overlap between genes with expression affected by bacteria and lack of SmD3-b protein. This supports the notion that *smd3b-1* mutation affects expression of a subset of genes whose mRNA levels normally change during bacterial attack. Analysis of enriched gene ontology (GO) terms showed that affected mRNAs are related to specific categories that are often common between sets (Figure 2B and Supplementary Dataset 2). As expected, bacterial treatment upregulated expression of genes involved in defense response and immune system processes in both Col-0 and the mutant. However, enrichment of genes in defense-related GO terms was clearly less prominent in the *smd3b-1* mutant than in wild-type plants (Figure 2B).

**FIGURE 3:**
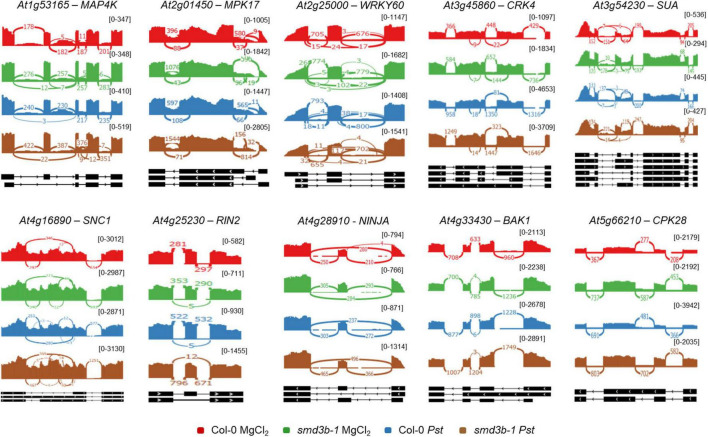
Both*Pst* infection and *smd3b-1* mutation affect alternative splicing (AS) events. Analysis of AS events for selected genes in control (MgCl_2_) and infected (48 hpi; *Pst*) Col-0 and *smd3b-1* plants. AS events are significantly altered for genes involved in pathogen response. Sashimi plots were created from RNA-seq data using Integrative Genomics Viewer (IGV). The numbers in brackets are the range on the bar graph. Note differences in scales for each sashimi plot.

The *smd3b-1* mutant showed altered expression of several genes from the defense response category, either in control or post-infection conditions (Supplementary Dataset 3 and Figure 2D and Supplementary Figure 5). Notably, a number of genes encoding key pathogenesis-related genes *PR3*, *PR4* and *PR5*, and plant defensin genes, *PDF1.2* and *PDF1.3*, as well as JA- and COR-induced *NATA1* (*N-ACETYLTRANSFERASE ACTIVITY 1*) (Lou et al., 2016), were markedly downregulated in the mutant in control conditions, but became strongly activated at later time points following *Pst* treatment (Supplementary Dataset 3, see Figures 1D,F). Similar effect was observed for genes of pathogenesis regulatory transcription factors *ANAC019, ANAC055* and *ANAC072* (*NAC DOMAIN CONTAINING PROTEIN*), and *WRKY70*. Another interesting observation in plants lacking SmD3-b is altered expression of factors that regulate BIK1 turnover, namely upregulation of *CPK28* (*CALCIUM-DEPENDENT PROTEIN KINASE 28*), *IRR* (*IMMUNOREGULATORY RNA-BINDING PROTEIN*) and *PERK1/2* (*PEP RECEPTORS*) genes, and strong downregulation of *PUB26* (*PLANT U-BOX 25/26*) E3 ligase (Supplementary Dataset 3). CPK28, a negative regulator of immune response, phosphorylates BIK1 and E3 ligases PUB25/PUB26 that target BIK1 for degradation. *CPK28* mRNA undergoes alternative splicing as a result of immune activation by Plant Elicitor Peptides (Peps), producing a retained-intron variant that encodes a truncated, inactive protein. This in turn leads to BIK1 stabilization and amplification of immune defense (Dressano et al., 2020). Noteworthy, the *smd3b-1* mutation results in accumulation of the retained-intron *CPK28* variant (Supplementary Dataset 4 and Figure 3). These observations suggest that the pathway involving Pep-induced BIK1 turnover could be generally enhanced in plants lacking SmD3-b, possibly contributing to modulation of disease resistance of the mutant.

Antibacterial defense is regulated by biotic stress hormones SA, JA and ABA that are involved in controlling stomatal movement, MAPK signaling, generation of reactive oxygen species and stimulation of callose deposition. The expression of several factors of the SA-JA and ABA signaling pathways was altered in the *smd3b-1* mutant in control conditions or during *Pst* infection (Supplementary Dataset 3, see Figure 2B and Supplementary Figure 5). Among the most important changes was elevated expression of protein phosphatase *PP2C/HAI1* (*HIGHLY ABA-INDUCED PP2C 1*) in both control and post-infection conditions. PP2C/HAI1, induced by ABA and COR upon *Pst* infection, dephosphorylates MPK3 and MPK6 kinases leading to their inactivation and immune suppression (Mine et al., 2018). In turn, genes involved in SA synthesis and signaling were more strongly induced by *Pst* infection in *smd3b-1*. This concerns for example SA-synthesis genes *EDS5* (*ENHANCED DISEASE SUSCEPTIBILITY 5*), *PBS3* (*AVRPPHB SUSCEPTIBLE 3*) and *SID2/ICS1* (*SALICYLIC ACID INDUCTION DEFICIENT 2/ISOCHORISMATE SYNTHASE 1*), N-hydroxy pipecolic acid (NHP)-synthesis genes *FMO1* (*FLAVIN-DEPENDENT MONOOXYGENASE 1*) and *ALD1* (*AGD2-LIKE DEFENSE RESPONSE PROTEIN 1*) (Zhang and Li, 2019) as well as SA methyltransferase and methyl esterase genes *BSMT1* (*BA/SA CARBOXYL METHYLTRANSFERASE 1*) and *MES9* (*METHYL ESTERASE 9*) (Attaran et al., 2009; Liu et al., 2011). Other relevant differences between *smd3b-1* and Col-0 plants related to the hormonal crosstalk include altered activation of ABA biosynthesis gene *NCED3* (*NINE-CIS-EPOXYCAROTENOID DIOXYGENASE 3*), upregulated expression of JA biosynthesis enzyme *LOX2* (*LIPOXYGENASE 2*) and negative transcriptional repressors of the JA-responsive genes, *JAZ1*, *JAZ5* and *JAZ9* (Zhang L. et al., 2017).

Changes in gene expression were confirmed by RT-qPCR for six selected defense response-related genes (Figure 2C). As seen previously (see Figures 1D,F), *smd3b-1* mutation resulted in a significant decrease of *PDF1.2* and *PR4* mRNAs under normal conditions, whereas expression of *FLS2*, which is required for the perception of PAMP flagellin, was markedly increased. In turn, after *Pst* treatment *SID2/ICS1*, *PR4* and *EDS5* showed significant upregulation in the mutant. These results, as well as our northern blot and RT-qPCR analyses (see Figures 1D,F and Supplementary Figures 1D,E) were consistent with RNA-seq results (see Figure 1G), so altered expression of pathogen markers in the *smd3b-1* mutant before and after *Pst* treatment was confirmed by three different methods. These observations suggest that the *smd3b-1* mutant shows perturbations in defense response to bacteria, including regulation of the immune system and response to ROS and biotic stress hormones. Both *Pst* treatment and *smd3b-1* mutation affected expression of genes involved in other numerous processes including histone modification, DNA replication, cell cycle, Golgi apparatus, chloroplast stroma and thylakoid. Another interesting observation was that pathogen treatment of the mutant appeared to decrease expression of genes involved in photosynthesis, chloroplast activity, and ribosome biogenesis and function (Figure 2D and Supplementary Figure 5). Northern hybridizations using probes located downstream of 18S and 5.8S rRNA revealed moderately altered level of 35S and 25SA/B rRNA precursors in the *smd3b-1* mutant upon *Pst* infection, confirming that pre-rRNA processing is indeed affected (Supplementary Figure 6). Still, the bases of these effects or their implications for bacterial infection are unclear.

Differences between *smd3b-1* and wild-type plants in expression of genes related to pathogenesis, both in control and post-infection conditions, are well illustrated by clustering analysis, where specific trends are clearly visible (Supplementary Figure 7). Of special interest is for example a large number of genes in response to bacterium and immune system GO terms that upon *Pst* infection are strongly upregulated in Col-0 but have a much lower final expression in the mutant (clusters 1, 2 in Supplementary Figure 7A). Another class represents genes in the same GO terms with *Pst*-induced expression in Col-0 that become even more highly activated in *smd3b-1* (cluster 3 in Supplementary Figure 7A). In turn, many genes that are downregulated in Col-0 in response to *Pst* often have decreased expression in *smd3b-1* plants already in control conditions and are affected by the pathogen to a lesser extent (clusters 1 in upper panel and 1, 2 in lower panel, Supplementary Figure 7B). The latter behavior is also observed for many photosynthesis- and chloroplast-associated genes (clusters 1, 2 in Supplementary Figure 7C). A general *Pst*-mediated suppression of these genes reflects the central role of chloroplasts in plant immunity as a major site for production of ROS and defense-related hormones SA, JA, and ABA (de Torres Zabala et al., 2015; Lewis et al., 2015; Serrano et al., 2016; Lu and Yao, 2018).

We also analyzed global changes in mRNA alternative splicing using rMATS (Shen et al., 2014), which allowed identification of AS events significantly altered during *Pst* infection or by the *smd3b-1* mutation (FDR < 0.05; ΔPSI (percent spliced-in) > 0.05; Table 1 and Supplementary Dataset 4). The highest number of affected splicing events was observed after *Pst* treatment in the mutant compared to the wild-type. In turn, comparing these two lines in control conditions identified fewer changes in the AS pattern, whereas *Pst* infection alone generated far less AS events in both wild-type and mutant plants. Based on the number of events and affected genes, we conclude that lack of SmD3-b protein has a greater impact on the splicing pattern than response to pathogenic bacteria. Nonetheless, it is apparent that splicing deficiency resulting from Smd3-b dysfunction is further exacerbated by pathogen infection. Interestingly, a high number of differential events for each comparison (from 25.3 to 33.4% depending on the set) was novel according to AtRDT2 annotation (Zhang R. et al., 2017). It appears that retained introns (RI) represented the most common AS events (from 55.3 to 71.8%), while alternative 5′ and 3′ spliced sites (A5/A3) or skipped exons (SE) were much less numerous (Table 1). Further assessment revealed that from 45.9 to 65.7% of splicing events and from 26.8 to 41.8% of genes with splicing events were unique when compared to other sets (Supplementary Figure 8A). As expected, lack of the core snRNP protein SmD3-b destabilizes U1, U2 and U4 snRNAs (Dataset S3 and Supplementary Figure 8B), supporting a general splicing defect in *smd3b* plants (Swaraz et al., 2011). Differential splicing events for six genes were visualized using Sashimi plots and verified by RT-qPCR (Supplementary Figure 9A). All of these genes showed changes in the splicing pattern due to *smd3b-1* mutation, however, splicing of *At5g20250* and *At5g57630* was also altered in wild-type plants during *Pst* infection. Treatment with flg22 had a similar impact on the splicing pattern in the mutant as the pathogen (Supplementary Figure 9B). These results suggest that both, early PAMP-induced and later pathogen-triggered, stages of infection affect SMD3-regulated splicing events. Among genes having significantly affected AS events in the mutant we found several genes involved in pathogen response, including *MAP4K* (*MITOGEN-ACTIVATED PROTEIN KINASE KINASE KINASE KINASE4*), *MPK17* (*MAP KINASE 17*), *WRKY60*, *CRK4* (*CYSTEINE-RICH RLK 4*), *SUA* (*SUPPRESSOR OF ABI3-5*), *SNC1*, *RIN2* (*RPM1 INTERACTING PROTEIN 2*), *NINJA* (*NOVEL INTERACTOR OF JAZ*), *BAK1*, *CPK28* and *SNC4*, *At3g44400*, *PRX34* (*PEROXIDASE 34*), *AGG1* (*HETEROTRIMERIC G PROTEIN GAMMA-SUBUNIT*), *MEKK3* (*MAPK/ERK KINASE KINASE* 3), *CRK6* (*CYSTEINE-RICH RLK* 6), *XLG2* (*EXTRA-LARGE G PROTEIN 3*) and *TGA2* (*TGACG SEQUENCE-SPECIFIC BINDING PROTEIN 2*) (Figure 3 and Supplementary Figure 9C, Supplementary Table 1, and Supplementary Dataset 4).

**TABLE 1:** Alternative splicing events and genes significantly altered by infection or *smd3b-1* mutation.

	Col-0 *Pst* vs Col-0 MgCl_2_	*smd3b-1 Pst* vs *smd3b-1* MgCl_2_	*smd3b-1* MgCl_2_ vs Col-0 MgCl_2_	*smd3b-1 Pst* vs Col-0 *Pst*
genes	1010 (217)	775 (234)	1685 (384)	1955 (495)
events	1261 (319)	961 (320)	2524 (705)	3025 (1012)
A3	158 (68)	118 (71)	336 (175)	344 (195)
A5	104 (1)	108 (4)	389 (22)	326 (21)
RI	906 (223)	640 (207)	1396 (378)	1844 (603)
SE	82 (17)	79 (28)	366 (99)	470 (159)
MX	11 (10)	16 (10)	37 (31)	41 (34)

*Abbreviation: A3, alternative 3′ splice site; A5, alternative 5′ splice site; RI, retained intron; SE, skipping exon; MX, mutually exclusive exons. The numbers in brackets represent novel alternative splicing events and genes.*

Global analysis of the transcriptome showed that *smd3b-1* mutation affected mRNA levels and splicing, including alternative splicing, both in normal conditions and during *Pst* infection. Since these changes concern several pathogenesis factors we conclude that this outcome directly or indirectly impacts the response to pathogen attack.

### Lack of SmD3 Dysregulates the Response to Biotic Effectors

Pathogen-associated molecular patterns directly activate the innate immune response in plants. Flg22 and elf18 are recognized by PRR receptors, which induce a pattern-triggered immunity response mediated by receptor-like kinases FRK1 (FLG22-INDUCED RECEPTOR-LIKE KINASE 1) and BAK1. These proteins activate the MAP kinase cascade, leading to the expression of defense genes, such as *WRKY* transcription factors. In turn, COR stimulates JA-signaling and in consequence suppresses SA-dependent defense (Brooks et al., 2005).

**FIGURE 4:**
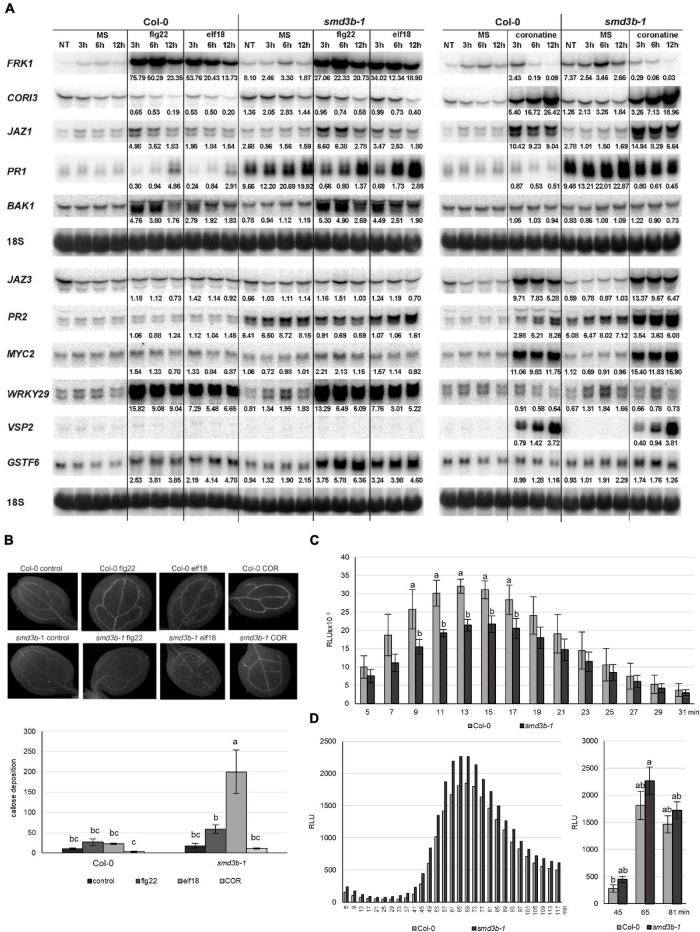
Pathogen-associated molecular patterns (PAMP)-induced expression of pathogenesis markers, callose deposition and production of ROS is altered in the *smd3b-1* mutant.**(A)** Northern blot analysis of factors involved in PAMP response. Samples were collected at indicated time points from non-treated (NT) 14-day -old seedlings, treated with MS (control) or 100 nM of flg22, elf18 and COR. The ratio of transcript level in treated Col-0 and *smd3b-1* relative to control (MS) and the ratio of control *smd3b-1* vs. Col-0 is shown as numbers. Values were normalized to 18S rRNA loading control. Experiments were repeated four times; representative blots are shown. **(B)** One-week-old plants were treated with MS (control) or 1 μM of flg22, elf18 and COR. Callose formation was visualized by aniline blue staining and epifluorescence microscopy and quantified using ImageJ software from digital photographs as a number of local maxima specified by the average of RGB colored pixels (callose intensity) in plant material. Bars represent mean of three independent biological replicates with error bars showing *SD*; letters represent significant difference (*P* < 0.05) for Tukey’s HSD test. Representative pictures are shown. **(C,D)** ROS production in response to 100 nM flg22 **(C)** or *Pst* **(D)** treatment in leaf disks from 6-week-old Col-0 and *smd3b-1* plants. **(C)** Bars represent mean of four independent biological replicates with error bars showing *SD*, with Student’s *t*-test. **(D)** Letters represent significant difference (*P* < 0.05) for Tukey’s HSD test. Luminescence is in Relative Light Units (RLUs).

To investigate the mode of action of PAMP-induced pathways in the *smd3b-1* mutant, we treated 14-day-old seedlings with flg22, elf18, or COR and examined selected mRNAs involved in biotic stress responses by northern blot (Figure 4A). In accordance with their biological activities, flg22- and elf18-induced expression of *FRK1*, *BAK1*, and *WRKY29* mRNAs, whereas COR treatment activated *JAZ1*, *JAZ3*, *MYC2* (*JASMONATE INSENSITIVE 1*), and *VSP2* (*VEGETATIVE STORAGE PROTEIN 2*) components of the MYC branch of the JA pathway in both wild-type and the mutant plants compared to control conditions. These results confirmed the specificity of flg22/elf18- and COR-mediated pathways, as their respective components were not affected by unrelated PAMPs. The pattern of pathogen markers in the *smd3b-1* mutant differed from that in wild-type plants, especially for *CORI3* (*CORONATINE INDUCED 1*), *PR1*, *PR2* and *FRK1*, which showed elevated basal expression, suggesting constitutive activation of stress response genes. Also, some transcripts were induced by PAMPs to varying extents in the mutant, e.g., activation of *BAK1* was stronger and *FRK1* weaker following flg22 or elf18 treatment, the levels of *GSTF6* and *JAZ1* were upregulated by flg22, while COR-triggered accumulation was lower for *CORI3* and slightly higher for *MYC2* (Figure 4A). Still, these changes were relatively modest and mainly led to the adjustment of the final levels in both mutant and wild-type.

These data confirm that flg22/elf18- and COR-induced responses represent separate pathways and show that SmD3-b only moderately affects PAMP-triggered activation of early- or/and late-responsive genes involved in plant innate immunity. Also, these observations are consistent with the contribution of COR to pathogenesis by suppressing basal defense-associated genes (Thilmony et al., 2006).

Another important indicator of PTI is PAMP-induced deposition of callose in cotyledons or leaves. To test effects of *smd3b-1* mutation on callose accumulation, the mutant and wild-type seedlings were treated with flg22, elf18, or COR effectors and examined by microscopy 24 h after treatment (Figure 4B). Quantification of the callose signal revealed that elf18- and flg22-induced deposition of callose was significantly higher in the *smd3b-1* leaves than in Col-0. As shown previously, callose production was suppressed by COR treatment (Geng et al., 2012). This result, suggesting that lack of SmD3 somehow promotes accumulation of callose, is rather counter-intuitive as callose is supposed to reinforce the cell wall against pathogen entry (Ellinger et al., 2013). However, callose and pathogen resistance is modulated by SA-dependent disease resistance and is affected by several factors, such as growth and stress conditions, thus callose deposition does not always match the activity of plant immunity (Nishimura et al., 2003; Luna et al., 2011).

*Pst* infection through PAMPs also elicits production of reactive oxygen species. The primary, low-amplitude, apoplastic ROS, dependent on cell wall peroxidases and plasma membrane NADPH oxidases, triggers PTI-dependent basal antimicrobial defense (Daudi et al., 2012; Shapiguzov et al., 2012; Jwa and Hwang, 2017). To test whether SmD3 is required for activation of the early PTI response, we measured the levels of flg22-triggered H_2_O_2_ in wild-type and *smd3b-1* plants. A luminol-based assay for leaves treated with flg22 revealed that ROS accumulation was significantly reduced in the mutant compared to Col-0 (Figure 4C). This effect may be due to attenuation of RBOHD (Respiratory Burst Oxidase Homolog D) activity. RBOHD is the major ROS-generating plasma membrane NADPH oxidase and is regulated by phosphorylation and ubiquitination (Kadota et al., 2015; Lee et al., 2020). One of the RBOHD-phosphorylating kinases, receptor-like cytoplasmic kinase PBL13 (PBS1-like kinase 13), acts as a negative regulator of RBOHD stability and activity (Lee et al., 2020). Expression of *PBL13* is markedly upregulated in the absence of SmD3-b (Supplementary Dataset 3) and this may lead to destabilization of RBOHD and decreased ROS production. Next, we also checked the production of the ETI-associated, high-amplitude, chloroplastic ROS after pathogen treatment and found out that, in contrast to the apoplastic ROS, a markedly higher level of the secondary intracellular ROS was generated in the *smd3b-1* mutant (Figure 4D). Recent studies have demonstrated that also the ETI-associated ROS burst is triggered by RBOHD and is enhanced by PTI (Ngou et al., 2021; Yuan et al., 2021). RBOHD is mainly phosphorylated by BIK1, its close homolog PBL1 and LecRK-I.9/DORN1 (DOESN’T RESPOND TO NUCLEOTIDES 1) kinases (Kadota et al., 2014; Li et al., 2014; Chen et al., 2017). We envisage that the more robust production of *Pst*-induced ROS in *smd3b-1* plants may be due to the less efficient BIK1 turnover and the increased expression of *DORN1* in the mutant (Supplementary Dataset 3). These results suggest that while a weaker burst of apoplastic ROS in the absence of SmD3-b may result in a less effective inhibition of pathogen multiplication, a stronger intracellular ROS accumulation possibly reinforces plant stress response by activation of defense-related genes.

Different stress conditions that involve ROS production evoke endonucleolytic cleavage of tRNA molecules at the anticodon loop (Thompson et al., 2008). tRNA fragments (tRFs) may contribute to translation inhibition during microbial attack or act as signaling molecules in the course of the stress response (Schimmel, 2018). To determine whether biotic stress in *Arabidopsis* also results in tRNA fragmentation, we checked decay intermediates for a few tRNAs in Col-0 and *smd3b-1* plants following treatment with *Pst* DC3000 at 24, 48, and 72 hpi (Supplementary Figure 10). As expected, bacterial infection led to accumulation of shorter RNA fragments indicative of tRNA cleavage. Interestingly, the amount of tRFs was increased in the mutant, in line with a higher ROS level. It is therefore possible that this outcome reflects a compromised defense of the mutant toward the pathogen.

Taken together, these results show that SmD3 modulates not only the early PTI but also late ETI responses. This is consistent with a recent notion that there is an extensive overlap and interplay between the these two stages of immune signaling (Ngou et al., 2021; Yuan et al., 2021).

### Lack of SmD3-b Affects Pathogen Entry

Our data on changes in mRNA levels of pathogenesis markers in *smd3b-1* plants following *Pst* infection are inconsistent with compromised resistance of the mutant. This suggests that the observed phenotype could be attributed to other factors related to plant immunity.

Sensitivity to the pathogen may also arise due to alterations in stomatal functioning since stomata play an important role in plant immunity as a major entryway for bacteria (Melotto et al., 2006; Cao et al., 2011; Lim et al., 2015). RNA-seq data revealed that a large number of genes that are involved in stomatal development, movement or dynamics were significantly changed in the *smd3b-1* mutant, in both control and post-infection conditions (Figure 2D and Supplementary Dataset 3). These included regulators of stomatal density and patterning (e.g., *STOMAGEN* (*EPFL9, EPIDERMAL PATTERNING FACTOR-LIKE 9*), *EPF2* (*EPIDERMAL PATTERNING FACTOR 2*), *TMM* (*TOO MANY MOUTHS*), *ER* (*ERECTA*) and *ERL1* (*ERECTA LIKE 1*) (Pillitteri and Torii, 2012; Simmons and Bergmann, 2016), ABA-induced stomatal closure (e.g., PP2C phosphates *ABI1* (*ABA-INSENSITIVE 1*), *ABI2* and *HAB1* (*HOMOLOGY TO ABI1 1*), ubiquitin E3 ligase *CHYR1*/*RZPF34* (*CHY ZINC-FINGER AND RING PROTEIN 1/RING ZINC-FINGER PROTEIN 34*), *GHR1* (*GUARD CELL HYDROGEN PEROXIDE- RESISTANT 1*), *SLAC1* (*SLOW ANION CHANNEL-ASSOCIATED 1*), *SIF2* (*STRESS INDUCED FACTOR 2*) (Hua et al., 2012; Lim et al., 2014; Ding et al., 2015; Guzel Deger et al., 2015; Lim and Lee, 2015; Chan et al., 2020), and stomatal reopening (e.g., NAC transcription factors *ANAC019, ANAC055* and *ANAC072*, SA synthesis and modification enzymes *SID2*/*ICS1* and *BSMT1* Zheng et al., 2012). Finally, altered were also the levels of *LecRK-V.5*, *LecRK-VI.2* and *LecRK-I.9* (*LEGUME-LIKE LECTIN RECEPTOR KINASES*) that act as negative or positive regulators of stomatal immunity, respectively (Desclos-Theveniau et al., 2012; Singh et al., 2012; Supplementary Dataset 3). Among genes involved in stomatal development and movement we also found several with significantly altered AS events in the mutant, including *ABCC5* (*ATP-BINDING CASSETTE C5*), *AKS2* (*ABA-RESPONSIVE KINASE SUBSTRATE 2*), *GRP7* (*GLYCINE RICH PROTEIN 7*), *GPA1* (*G PROTEIN ALPHA SUBUNIT 1*), *ER*, *CHYR1*/*RZPF34*, *TPK1* (*TWO PORE K CHANNEL 1*), and *HAI1* (Supplementary Figure 11, Supplementary Dataset 3). These observations show that SmD3 affects the level and alternative splicing pattern of transcripts related to stomata development and function.

**FIGURE 5:**
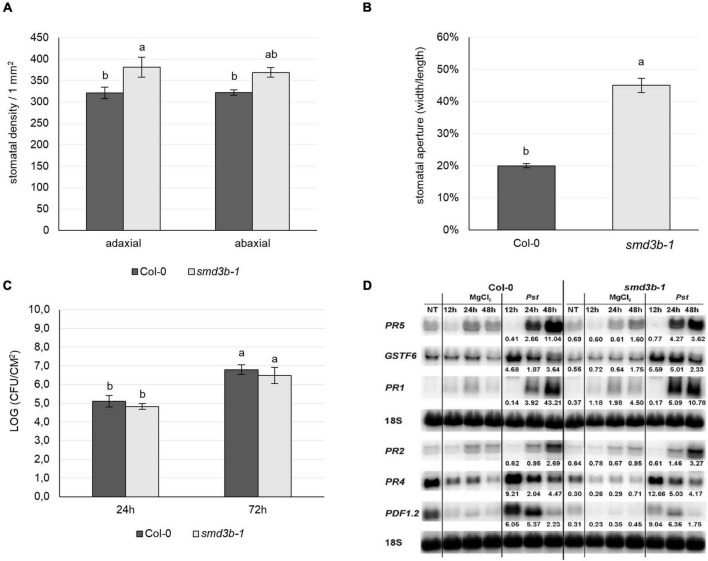
SmD3 contributes to stomatal immunity. **(A,B)** Stomatal density and aperture of Col-0 and *smd3b-1* plants. Comparison of the stomatal density on the abaxial and adaxial leaf side of Col-0 and *smd3b-1*, shown as number of stomata per 1 mm^2^ of leaf surface **(A)**. Stomatal aperture in Col-0 and *smd3b-1* **(B)**. The aperture on the abaxial leaf side was calculated as stomata width to length to ratio and expressed as percentage. **(C)** The *smd3b-1* mutant is not sensitive to *Pst* after infiltration inoculation. Growth of *Pst* DC3000 at 24 and 72 hpi after syringe infiltration in Col-0 and the *smd3b-1* mutant. For each time point leaf disks were collected from 8 plants. Results are mean of two independent experiments and error bars represent SD. **(A–C)** Letters represent significant difference (*P* < 0.05) for Tukey’s HSD test. **(D)** Northern blot analysis of factors involved in pathogen response. Samples were collected from non-treated (NT), control (MgCl_2_) and injected (*Pst*) Col-0 and *smd3b-1* plants at indicated time points. Numbers represent transcript level in *Pst*-treated Col-0 and the *smd3b-1* relative to control and normalized to 18S rRNA loading control.

We therefore checked the state of stomatal density and aperture in wild-type and *smd3b-1* plants and observed that both were significantly increased in the mutant (Figures 5A,B). Such features may enable faster entry and facilitate proliferation of bacteria in *smd3b-1* plants, and as a consequence increase sensitivity to pathogen. To confirm this possibility we used a different infection method, i.e., syringe infiltration, in which the pathogen is not delivered into the leaf tissue *via* stomata as in a natural situation but directly to the apoplastic space. The results showed that, as opposed to surface inoculation by spraying, *smd3b-1* plants were no longer sensitive to *Pst* after infiltration inoculation (Figure 5C). Moreover, also in stark contrast to spraying, induction of key pathogenesis markers (*PR1*, *PR2*, *PR4*, *PR5*, *PDF1*.2 and *GSTF6*) after 48hpi was similar or even weaker in the *smd3b-1* mutant compared to Col-0 (Figure 5D). This result is consistent with observations that *Pst*-induced changes in gene expression in the mutant are more prominent than these triggered by PAMPs (see Figures 1D,E, 4A). Such an outcome may reflect a situation when the pre-invasive stage of defense response, i.e., pathogen entry, is affected in the mutant. Since pathogen entry to the apoplastic space is restricted by PAMP-induced stomatal closure mediated by ABA signaling in guard cells, we next tested stomatal movement following ABA treatment. There was virtually no difference in ABA-stimulated stomatal closure between wild-type and *smd3b-1* plants (Supplementary Figure 12), indicating that this aspect of defense signaling was not impaired in the mutant. Still, the higher number of stomata and their larger pores in the *smd3b-1* probably lead to initial more unrestrained pathogen entry, thereby increasing sensitivity to *Pst*. Notably, higher stomatal density together with impaired stomatal closure of mutants in MAPK kinases, MPK3, MPK6, MKK4 and MKK5, have been demonstrated to compromise stomatal immunity, leading to their enhanced susceptibility (Su et al., 2017). Together these data suggest that proper functioning of SmD3b contributes to establishing effective stomatal immunity.

### SmD3-b Modifies Levels of pri-microRNAs and Mature MicroRNAs Upon *Pst* Infection

**FIGURE 6:**
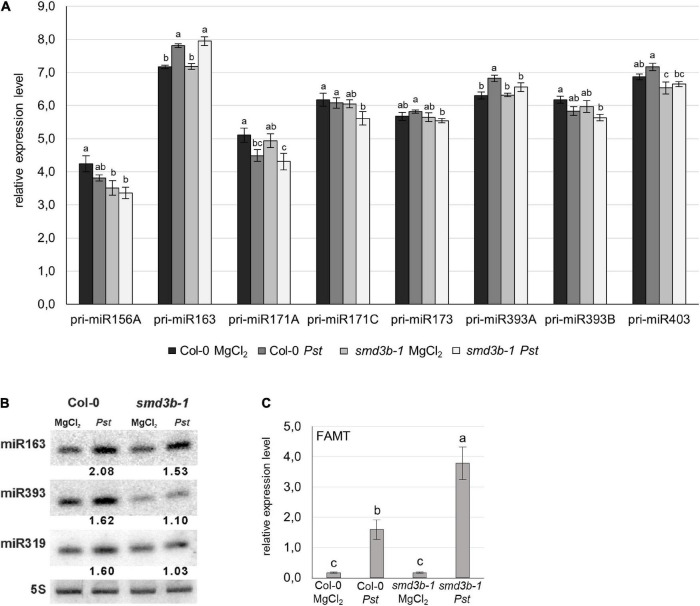
Both*smd3b-1* mutation and *Pst* infection modify the level of pri-miRNA and mature miRNA.**(A)** RT-qPCR analysis of chosen pri-miRNAs during *Pst* infection. Samples were collected from 6-week-old control (MgCl_2_) and infected (48 hpi, *Pst*) Col-0 and *smd3b-1* plants. Results represent mean of three independent biological replicates with error bars showing *SD*; letters represent significant difference (*P* < 0.05) for Tukey’s HSD test.*GAPDH* mRNA was used as a reference. **(B)** Northern blot analysis of miRNAs involved in pathogen response. Numbers represent the levels of miRNA in *Pst*-treated Col-0 and the *smd3b-1* mutant relative to control (MgCl_2_), normalized to 5S rRNA loading control. Experiments were repeated at least three times; representative blots are shown. **(C)** RT-qPCR analysis of miR163 target, *FAMT* mRNA. Mean values ± SEM were obtained from three independent experiments; letters represent significant difference (*P* < 0.05) for Tukey’s HSD test. *UBC9* mRNA was used as a reference.

Since plant miRNAs are differentially expressed during pathogen infection and may contribute to the regulation of plant immunity we evaluated changes in pri-miRNA and miRNA levels in *Pst*-infected 6-week-old *smd3b-1* and Col-0 plants (48 hpi). RT-qPCR analysis showed significant changes for 8 out of 14 tested pri-miRNAs (Figure 6A and Supplementary Figure 13). In the control condition, the level of pri-miR156A and pri-miR403 was reduced in the mutant compared to the wild-type, whereas expression of pri-miR171A, pri-miR171C, and pri-miR393B was downregulated following pathogen treatment. In contrast, accumulation of pri-miR163 and pri-miR393A was increased after *Pst* infection in both mutant and wild-type plants. The expression of corresponding mature miRNAs was analyzed by northern blot (Figure 6B). We observed that after *Pst* treatment miR163, miR393, and miR319 were upregulated to a lesser extent in the *smd3b-1* than in wild-type plants. We also tested the level of *FAMT* mRNA, which is one of the targets of miR163 and has a direct role in pathogen response (Chow and Ng, 2017). In agreement with accumulation of miR163, the induction of *FAMT* mRNA 48 hpi was significantly stronger in the mutant compared to the wild-type (Figure 6C). These results suggest a correlation between expression of miRNAs and their targets to modulate defense response against pathogen.

## Discussion

**FIGURE 7:**
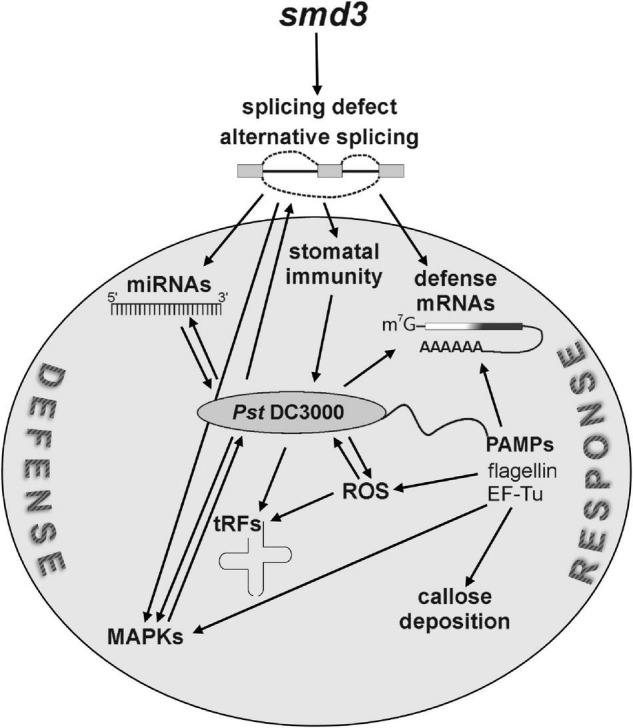
SmD3 affects several aspects of plant immunity through regulation of splicing of key pathogenesis factor mRNAs. Splicing defects in *smd3* mutants impact stomatal immunity, induction of pathogen-related proteins, including WRKY transcription factors, MAPKs cascade signaling, ROS production and miRNA-dependent fine-tuning of plant defense.

Pre-mRNA splicing, especially alternative splicing, regulates many different cellular and physiological processes in plants, such as development, signal transduction and response to environmental cues, including biotic stress caused by microbial attack (Yang et al., 2014; Meyer et al., 2015). Still, despite realization that the majority of expressed genes in *Arabidopsis* undergo alternative splicing upon *P. syringae* infection (Howard et al., 2013), the extent of this level of regulation has not been extensively evaluated. We assessed long-term effects of splicing deficiency on plant immunity and we showed that a general splicing defect in a core spliceosomal component mutant, *smd3b-1*, results in decreased resistance to virulent *Pst* DC3000. Similar effects on pathogen proliferation were also observed for the *smd3a* knock-out and *smd1b* mutant in another spliceosomal core protein, suggesting the involvement of the whole Sm complex. Our analyses reveal that spliceosome dysfunction impacts several aspects of pathogen response, namely stomatal immunity, activation of resistance-related factors and pathogen-associated WRKY transcription factors, ROS production and miRNA-dependent fine-tuning of plant defense (Figure 7). Our global transcriptome analysis of Col-0 and *smd3b-1* plants showed that *smd3b-1* mutation affects mRNA levels and splicing pattern both in normal conditions and during *Pst* infection. Our data also adds to the description of transcription- and splicing-mediated reprogramming of gene expression caused by pathogen-induced stress in plants.

### Transcriptome Upon *Pst* Infection Shapes *smd3b-1* Defense Response

High-throughput RNA-seq analyses of transcriptome dynamics in *Arabidopsis* plants following infection with virulent DC3000 or ETI-triggering avirulent *Pst* strains (AvrRpt2 and AvrRpm1) showed that transcriptional response to avirulent pathogens was really fast, already observed at 4 hpi, whereas the equivalent response to virulent *Pst* was much slower and reached the same level at 24 hpi (Mine et al., 2018). Although we focused on the long-term response (48 hpi) to virulent *Pst* DC3000 in Col-0 and the *smd3b-1* mutant to assess changes resulting from splicing defects, in line with previous reports we observed upregulated expression of many genes that belong to GO terms related to defense response and downregulation of chloroplast-associated genes (de Torres Zabala et al., 2015; Lewis et al., 2015; Mine et al., 2018). Changes in these ontology categories are characteristic of pathogen response as they include many genes that are key regulatory factors of plant immunity. We also note that the number of splicing events and genes with splicing events were increased following pathogen treatment, as reported previously (Howard et al., 2013). This enhancement of alternative splicing associated with pathogenic infection further underlines the correlation between these two processes. Transcriptome profiles of *smd3b-1* control and *Pst*-treated plants revealed a complex picture of fluctuations in the expression pattern of genes related to different aspects of plant immunity (see below). Among these the most spectacular is a strong downregulation of a number of genes encoding key *PR* factors in the mutant in control conditions as well as enhanced activation of pathogenesis markers following bacterial infection (Supplementary Dataset 3). Plants lacking SmD3-b also exhibit altered expression of pathogenesis regulatory transcription factors as well as components of the BIK1 degradation pathway. In addition, our RNA-seq data revealed that *smd3b-1* mutation affected the splicing pattern of several of pathogen response-related factors (see Figure 3 and Supplementary Figure 9 and Supplementary Table 1). We envisage that the resultant of these changes, including variation in AS events, may contribute to dysregulated response to pathogen and its effectors in the *smd3b-1* mutant.

Although regulation of plant immunity in *Arabidopsis* by pre-mRNA splicing has been reported for several splicing factors (Yang et al., 2014; Meyer et al., 2015), our analyses present evidence supporting such a role for the core spliceosome. Sm proteins interact physically and functionally with pICln and PRMT5 components of the methylosome complex that mediates snRNP assembly and the spliceosome activating nineteen complex (NTC) (Deng et al., 2016). Considering that these factors act as negative and positive regulators of plant immunity, respectively (Palma et al., 2007; Monaghan et al., 2009, 2010; Xu et al., 2011, 2012; Huang et al., 2016), their combined action in controlling disease resistance signaling *via* modulation of splicing is a strong possibility.

### Lack of SmD3-b Impacts the Pre-invasive Stage of Defense Response and May Lead to Enhanced Systemic Acquired Resistance and Defense Priming

Stomata are an integral part of the plant immune system and regulation of their aperture prevents pathogen entry into leaves and subsequent colonization of host tissues and disease symptoms. The *smd3b-1* mutation results in altered expression of a whole set of genes involved in stomata development and movement (Supplementary Dataset 3), including positive and negative regulators of stomatal density and patterning. Moreover, increased stomatal density and aperture in *smd3b-1* plants together with their sensitivity to pathogen delivered *via* stomata suggest that SmD3b dysfunction impacts mainly the pre-invasive stage of defense response.

Another aspect of bacterial propagation is related to stomatal dynamics during infection. Briefly, PAMP-triggered stomatal closure to restrict pathogen entry, followed by SA-dependent basal defense, are suppressed by *Pst* effectors and phytotoxin coronatine that activates the antagonistic JA pathway and leads to stomatal reopening (Melotto et al., 2008; Luna et al., 2011; Geng et al., 2014). Although expression of several genes responsible for stomatal movement is altered in *smd3b-1* plants, stomatal closure appears not be compromised, probably due to the opposing impact of the *smd3b-1* mutation on expression of these genes. On the other hand, *Pst*-induced stomatal reopening could be affected in plants lacking Smd3-b due to the enhanced activation of NAC transcription factors that are induced by COR, leading to the COR-mediated stomatal reopening and thus more effective pathogen penetration.

In addition, *smd3b-1* mutation may affect SAR and defense priming that protect uninfected parts of the plant against secondary infections by a broad spectrum of pathogens and activate a faster and more robust response (Fu and Dong, 2013; Ádám et al., 2018; David et al., 2019). First of all, genes involved in the synthesis and modification of SA and NHP (e.g., *EDS5, PBS3*, *SID2/ICS1, FMO1*, *ALD1, BSMT1* and *MES9/SABP2*) that are important regulators of SAR and defense priming are strongly induced by *Pst* infection in *smd3b-1* (Supplementary Dataset 3). Notably, the expression of some of these genes (e.g., *SID2/ICS1* and *PBS3*) is regulated by WRKY46, WRKY53 and WRKY70 transcription factors (Wang et al., 2006), which are also upregulated in the mutant following infection. In turn, the level of *NRT2* (*NITRATE TRANSPORTER 2*) after *Pst* treatment is markedly decreased in the mutant, but not in the wild-type, and this may lead to constitutive priming (Camañes et al., 2012). Finally, defense priming and SAR also depend on ROS generation and callose deposition (Conrath et al., 2015; Mauch-Mani et al., 2017), and these are enhanced in PAMP-treated mutant plants. These observations suggest that in the absence of SmD3-b both SAR and priming defense may be enhanced, possibly to counteract the compromised stomatal immunity.

### Photosynthesis and Chloroplast-Associated Genes in *smd3b-1* Plants

Normally, PAMP perception leads to a general suppression of nuclear encoded chloroplastic genes and inhibition of photosynthetic processes, leading to reactive oxygen burst and defense response (de Torres Zabala et al., 2015; Lewis et al., 2015; Serrano et al., 2016; Lu and Yao, 2018). Our clustering analysis revealed that these genes were indeed strongly downregulated in Col-0 in response to *Pst* (Supplementary Figure 7). Surprisingly, their expression was often decreased by *smd3b-1* mutation alone and was not further modified by pathogen attack (Figure 2, and Supplementary Figures 6, 8 and Supplementary Dataset 3). Such a situation may alter downstream events in the response pathway. Consistently, production of photosynthesis-derived, chloroplastic ROS was more robust in plants lacking SmD3-b, probably resulting in a stronger induction of many defense response genes (Figure 2 and Dataset S3). On the other hand, the primary apoplastic ROS burst was less pronounced in *smd3b-1* than in wild-type plants (Figure 4C), possibly as a result of RBOHD attenuation. Additional changes in the apoplastic oxidative burst could stem from deregulation of splicing of other factors involved in ROS production and signaling (Kadota et al., 2015; Qi et al., 2017; Waszczak et al., 2018). Indeed, in *smd3b-1* plants several genes, such as *BAK1*, *XLG2*, *PRX34*, *AGG1*, *CRK4* and *CRK6*, showed statistically significant changes in the pattern of AS events that in particular apply to a higher number of retained introns (see Figure 3 and Supplementary Figure 9C).

From our analyses of the impact of SmdD3-b dysfunction on plant defense emerges a pattern whereby the initial response, including compromised stomatal immunity and limited production of the PAMP-triggered apoplastic ROS, leads to increased susceptibility to bacterial infection. This is then followed by changes aiming at reinforcing plant defense systems through a more robust production of chloroplastic ROS, intensified hormonal signaling, enhanced callose deposition and stronger activation of defense-related genes. The interplay between these elements results in a complex and often opposing output of mutant defense response. Most importantly, this behavior accompanies surface inoculation of the pathogen that closely resembles a natural infection, and does not take place when bacteria are artificially infiltrated into the leaf intercellular space.

The *smd3b* mutant displays a range of physiological phenotypes, including impaired root growth, altered number of floral organs and late flowering (Swaraz et al., 2011). These phenotypes correlate well with extensive changes in gene expression and differences in the splicing pattern of a few hundred of pre-mRNAs. It is tempting to speculate whether there are connections between these morphological and molecular phenotypes and dysregulation of the response to bacterial pathogen, considering that similar effects were observed for several other *Arabidopsis* mutants with defects in pre-mRNA splicing or other RNA metabolic pathways. It is possible that defective RNA processing impacts development, hormone signaling and general fitness of the plant. These processes are closely coordinated with stress response pathways, including pathogen response, so the final result will reflect their interplay. Good examples of this phenomenon are provided by mutants in mRNA nonsense-mediated decay (*upf1* and *smg7*) or mRNA decapping (*pat1*), which exhibit autoimmunity and constitutive activation of plant defense leading to developmental aberrations (Riehs-Kearnan et al., 2012; Roux et al., 2015). In the case of SMD3-b, we envisage a somehow reverse situation, whereby its dysfunction primarily impacts stomatal immunity leading to increased susceptibility to pathogen. To counteract this drawback mutant plants may enhance pathogen response *via* adjusting the expression of key pathogenesis-related genes, possibly through alternative splicing. Based on the observed phenotypes we postulate that SMD3-b plays an important role not only in pre-mRNA splicing and spliceosome assembly but also acts as an intricate regulator of the plant defense response.

## Data Availability Statement

The original contributions presented in the study are publicly available. This data can be found here: National Center for Biotechnology Information (NCBI) BioProject database under accession number GSE117077.

## Author Contributions

AG designed and performed most of the experiments and conceived the project and wrote the manuscript with MK contributions. MK analyzed the RNA-seq results. MK, MS, JP, and JD performed some of the experiments. AJ and ZS-K supervised JD. JK supervised and completed the writing. All authors contributed to the article and approved the submitted version.

## Conflict of Interest

The authors declare that the research was conducted in the absence of any commercial or financial relationships that could be construed as a potential conflict of interest.

## Publisher’s Note

All claims expressed in this article are solely those of the authors and do not necessarily represent those of their affiliated organizations, or those of the publisher, the editors and the reviewers. Any product that may be evaluated in this article, or claim that may be made by its manufacturer, is not guaranteed or endorsed by the publisher.

## Footnotes

1
http://www.bioinformatics.babraham.ac.uk/projects/fastqc/

2
http://hgdownload.cse.ucsc.edu/admin/exe/linux.x86_64/

## Supplementary Material

Supplementary Figure 1 The *smd3b* and *smd3a* mutations cause changes in response to infection. **(A)** Structure of the *AtSMD3-a* (*At1g76300*) and *AtSMD3-B* (*At1g20580*) genes. Exons are represented by gray bars, UTRs are illustrated by black bars and localization of T-DNA insertions are indicated. **(B)** Growth of *Pst* DC3000 after 24 and 72 hpi in Col-0, *smd3a-2* and *smd3b-2* mutants. For each time point leaf disks were collected from 5 plants. Results are mean of two independent experiments. **(C)** Disease symptoms in Col-0 and *smd3b-1* 6-week-old plants (72 hpi). Experiments were repeated at least four times; representative pictures are shown. **(D)** Northern blot analysis of factors involved in pathogen response (another biological replicate). Samples were collected from non-treated (NT), control (MgCl_2_) and infected (*Pst*) Col-0 and *smd3b-1* plants at indicated time points. Numbers represent transcript level in *Pst*-treated Col-0 and the *smd3b-1* relative to control and normalized to 18S rRNA loading control. (E) RT-qPCR analysis of selected genes involved in pathogen response. Mean values ± SEM were obtained from three independent experiments, letters represent significant difference (*P* < 0.05) for Tukey’s HSD test. *UBC9* mRNA was used as a reference.

Supplementary Figure 2 *Pst*-induced expression of pathogenesis markers in the *smd3a-2* and *smd3b-2* mutants. Northern blot analysis of factors involved in pathogen response. Samples were collected from non-treated (NT), control (MgCl_2_) and infected (*Pst*) Col-0, *smd3a-2* and *smd3b*-2 plants at indicated time points. Numbers represent transcript level in *Pst*-treated Col-0, *smd3a-2* and *smd3b*-2 relative to control and normalized to 18S rRNA loading control.

Supplementary Figure 3 Both *smd1b* and *smd3b-1* mutants are susceptible to *Pst* infection. **(A)** Growth of *Pst* DC3000 after 24 and 72 hpi in Col-0, *smd1b* and *smd3b-1* mutants. For each time point leaf disks were collected from 5 plants. Results are mean of two independent experiments and error bars represent SD; letters represent significant difference (*P* < 0.05) for Tukey’s HSD test. **(B)** Northern blot analysis of factors involved in pathogen response. Samples were collected from non-treated (NT), control (MgCl_2_) and infected (*Pst*) Col-0 and *smd1b* plants at indicated time points. Numbers represent the ratio of transcript level in *Pst-*treated Col-0 and *smd1b* relative to control and normalized to 18S rRNA loading control.

Supplementary Figure 4 Results of RNA-seq analysis. **(A)** MA-plots (log_2_FC as a function of mean expression level on a logarithmic scale) of sequencing results for different comparisons, statistically significant hits (FDR < 0.05) are shown in red. Red numbers denote genes with significantly changed expression in each subgroup: up- or down-regulated, with absolute log_2_FC < 1 or > 1. **(B)** PCA (Principal component analysis) shows that biological replicas in RNA-seq create four groups based on the presence of the *smd3b-1* mutation and *Pst* infection.

Supplementary Figure 5 Both *Pst* infection and *smd3b-1* mutation impact mRNA expression. Bacterial infection and the *smd3b-1* mutation impact gene expression in selected categories. Volcano plots (−log_10_FDR as a function of log_2_FC) for genes implicated in DNA replication, cell cycle, histone modification, Golgi apparatus, response to jasmonic acid (JA) and salicylic acid (SA) and encoding components of ribosome. Red dots and triangles mark genes with significant changes.

Supplementary Figure 6 SmD3-b affects pre-rRNA processing. Northern blot analysis of rRNA precursors and intermediates in Col-0 and the *smd3b-1* mutant. Samples were collected from 6-week-old control (MgCl_2_) and infected (48 hpi, *Pst*) plants. rRNA precursors and intermediates are described on the right; molecular weight of 35S, 25S and 18S rRNA species are on the left. The position of specific *p5, p3*, 25S and 18S probes used for hybridization is shown in the diagram below. Numbers represent the ratio of the level of individual rRNA species in *Pst-*treated Col-0 and *smd3b-1* relative to control and normalized to *eIF-4A* mRNA loading control. Asterisk indicates cross-hybridization to organellar rRNA detected with probe *p3*.

Supplementary Figure 7 Differences between *smd3b-1* and Col-0 plants in expression of genes related to pathogenesis by clustering analysis. Clustering of gene expression profiles for genes affiliated with “response to bacterium,” “immune system process” **(A,B)**, “photosynthesis” and “chloroplast part” **(C)** GO terms that were significantly upregulated and downregulated after pathogen treatment. Number of genes in each cluster are depicted on each diagram. Clustering was performed using standard R functions.

Supplementary Figure 8 Splicing events and genes with splicing events significantly altered in Col-0 and the *smd3b-1* mutant upon *Pst* treatment. **(A)** Venn diagrams indicating the number of significant overlapping splicing events and genes with splicing events in Col-0 and the *smd3b-1* mutant after *Pst* treatment. Percent of unique splicing events and genes with splicing events when compared to other sets is given in parentheses. **(B)** *smd3b-1* mutation confers global splicing defect. U1 snRNA is destabilized in the absence of SmD3-b. Northern blot analysis of U1 snRNA. Samples were collected from non-treated (NT), control (MgCl_2_) and injected (*Pst*) Col-0 and *smd3b-1* plants at indicated time points. Numbers represent transcript level in Col-0 and *smd3b-1* relative to control and normalized to 5S rRNA loading control.

Supplementary Figure 9 *Pst*, flg22 treatment and *smd3b-1* mutation affect alternative splicing (AS) events. **(A)** Analysis of AS events by RT-qPCR for selected genes in control (MgCl_2_) and infected (48 hpi; *Pst*) Col-0 and *smd3b-1* plants; the type of AS event is described below each graph: SE- skipped exon, RI- retained intron, A5/A3- alternative 5′ or 3′ splice sites, respectively. Results represent mean of three independent biological replicates with error bars showing *SD*; **P* < 0.05; ***P* < 0.01; ****P* < 0.001 (Student’s *t*-test). *GAPDH* mRNA was used as a reference. Sashimi plots were created from RNA-seq data using Integrative Genomics Viewer (IGV). The numbers in brackets are the range on the bar graph. Note differences in scales for each sashimi plot. **(B)** Analysis of AS events by RT-qPCR for selected genes in control- (MS) and flg22-treated (100 nM for 6 h) Col-0 and *smd3b-1* plants. The type of AS event is described below each graph: SE- skipped exon, A3- alternative 3′ splice sites, respectively. Results represent mean of three independent biological replicates with error bars showing *SD*; **P* < 0.05; ***P* < 0.01; ****P* < 0.001 (Student’s *t*-test). *GAPDH* mRNA was used as a reference. **(C,D)** Analysis of AS events for selected genes in control (MgCl_2_) and infected (48 hpi; *Pst*) Col-0 and *smd3b-1* plants. AS events are significantly altered for genes involved in pathogen response. Sashimi plots were created from RNA-seq data using Integrative Genomics Viewer (IGV). The numbers in brackets are the range on the bar graph. Note differences in scales for each sashimi plot.

Supplementary Figure 10 *Pst* DC3000 infection increases production of tRNA fragments in the *smd3b-1* mutant. Northern blot analysis (PAGE) of three tRNA species following the *Pst* treatment. Samples were collected from non-treated (NT), control (MgCl_2_) and infected (*Pst*) Col-0 and *smd3b-1* plants at indicated time points. 7SL was used as a loading control. Asterisks indicate accumulation of short RNA fragments (tRFs). Experiments were repeated twice with similar results; representative blots are shown.

Supplementary Figure 11 *Pst* treatment and *smd3b-1* mutation affect alternative splicing (AS) events in genes involved in stomatal development and movement. Analysis of AS events for selected genes in control (MgCl_2_) and infected (48 hpi; *Pst*) Col-0 and *smd3b-1* plants. AS events are significantly altered for genes involved in pathogen response. Sashimi plots were created from RNA-seq data using Integrative Genomics Viewer (IGV). The numbers in brackets are the range on the bar graph. Note differences in scales for each sashimi plot.

Supplementary Figure 12 Stomatal closure following ABA treatment in Col-0 and *smd3b-1*. Mean values (± SEM) were obtained from six independent experiments with 40 stomata per data point; letters represent significant difference (*P* < 0.05) for Tukey’s HSD test.

Supplementary Figure 13 *Pst* infection changes the level of pri-miRNAs in the *smd3b-1* mutant. RT-qPCR analysis of chosen pri-miRNAs during *Pst* infection. Samples were collected from 6-week-old control (MgCl_2_) and infected (48 hpi, *Pst*) Col-0 and *smd3b-1* plants. Results represent mean of three independent biological replicates with error bars showing *SD* with no statistically significant changes. *GAPDH* mRNA was used as a reference.

Supplementary Table 1 Characteristics of genes involved in pathogen response having significantly affected alternative splicing events in the *smd3b-1* plants.

Supplementary Table 2 List of primers used in this study.

Supplementary Dataset 1 List of significantly affected genes based on RNA-seq.

Supplementary Dataset 2 Significantly enriched Gene Ontology terms among different gene sets.

Supplementary Dataset 3 List of genes involved in different aspect of pathogen response with significantly changed expression in the *smd3b-1* mutant.

Supplementary Dataset 4 List of alternative splicing events.
