# Supplementary material for: Arabidopsis Spliceosome Factor SmD3 Modulates Immunity to Pseudomonas syringae Infection
Source: Front Plant Sci. 2021 Dec 3;12:765003. doi: 10.3389/fpls.2021.765003 (PMC8678131; doi:10.3389/fpls.2021.765003)
Supplement: Supplementary Figure 1 — The smd3b and smd3a mutations cause changes in response to infection. (A) Structure of the AtSMD3-a (At1g76300) and AtSMD3-B (At1g20580) genes. Exons are represented by gray bars, UTRs are illustrated by black bars and localization of T-DNA insertions are indicated. (B) Growth of Pst DC3000 after 24 and 72 hpi in Col-0, smd3a-2 and smd3b-2 mutants. For each time point leaf disks were collected from 5 plants. Results are mean of two independent experiments. (C) Disease symptoms in Col-0 and smd3b-1 6-week-old plants (72 hpi). Experiments were repeated at least four times; representative pictures are shown. (D) Northern blot analysis of factors involved in pathogen response (another biological replicate). Samples were collected from non-treated (NT), control (MgCl2) and infected (Pst) Col-0 and smd3b-1 plants at indicated time points. Numbers represent transcript level in Pst-treated Col-0 and the smd3b-1 relative to control and normalized to 18S rRNA loading control. (E) RT-qPCR analysis of selected genes involved in pathogen response. Mean values ± SEM were obtained from three independent experiments, letters represent significant difference (P < 0.05) for Tukey’s HSD test. UBC9 mRNA was used as a reference. [file Data_Sheet_1.zip › data sheet 1/Presentation 1.pdf]

Supplementary Information – Materials and Methods

Analysis of stomatal density and aperture size

The experiments were performed using fully developed rosette leaves of 35-day-old *Arabidopsis* plants. The cleared epidermal peels from abaxial leaf surfaces and nail polish immersions from adaxial leaf surfaces were prepared and examined with a light microscope equipped with a Nikon Eclipse Ti camera, DS-Fi1c-U2 optics and Plan Apo VC 20x DIC N2. The counts were made on 6 leaves from independent plants for each ecotype and then averaged. For the measurements of stomatal aperture preparations were made from epidermal peels from abaxial leaf surfaces. Before preparation the leaves were incubated in a buffer composed of 10 mM KCl, 0.1 mM EGTA and 10 mM MES-KOH at pH 6.15 for 2 hours. Measurements were analysed using a light microscope, Eclipse Ti camera, DS-Fi1c-U2 optics and Plan Apo VC 20x DIC N2 (Nikon,Tokyo, Japan). The ratio of the length and width of the pore between guard cells was calculated using the NIS-Element program. The counts were made on 6 leaves from independent plants.

Bacterial infection assays by injection

Bacterial infection assays were performed with virulent *Pseudomonas syringae* pv. *tomato* strain DC3000 (*Pst*). Bacteria for inoculation were grown overnight in LB medium with rifampicin (50 µg ml^{-1}) at 28°C, resuspended in 10 mM $MgCl_2$ with density adjusted to 10^5 cfu ml^{-1} (OD_{600nm} = 0.003). 6-week-old plants were inoculated by injection with *Pst* suspension in 10 mM $MgCl_2$ and covered with plastic lids overnight. Material was harvested from at least 8 plants for each time point, frozen in liquid nitrogen and used for RNA extraction. Bacterial growth was quantified as the number of dividing bacterial cells 24 and 72 h after infection (hpi). Samples (four leaf discs) were taken using a cork-borer (4 mm) from 2 leaves per six plants in each independent replicate.
