# Supplementary material for: Arabidopsis Spliceosome Factor SmD3 Modulates Immunity to Pseudomonas syringae Infection
Source: Front Plant Sci. 2021 Dec 3;12:765003. doi: 10.3389/fpls.2021.765003 (PMC8678131; doi:10.3389/fpls.2021.765003)

Supplementary Figure S1

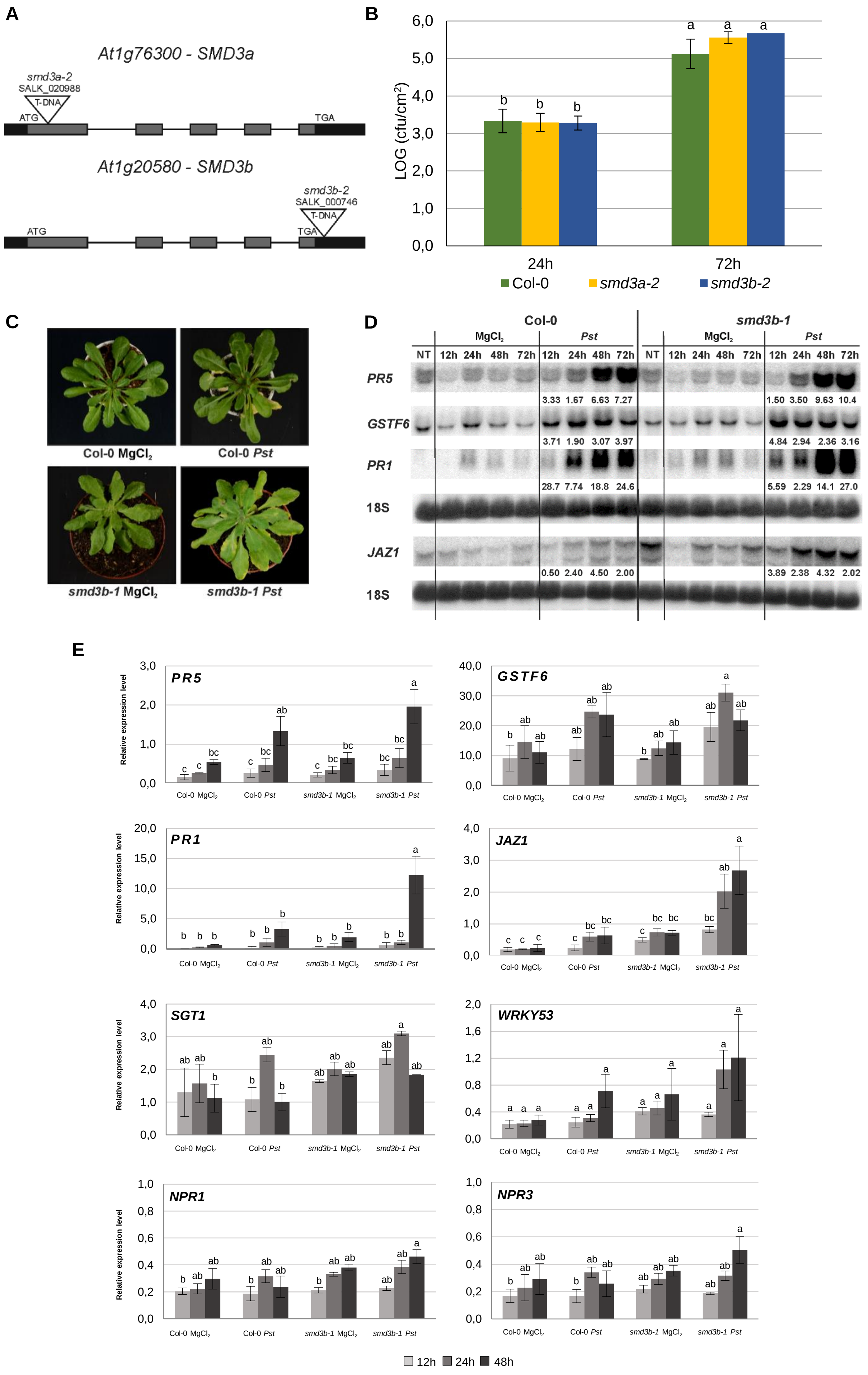

Supplementary Figure S2

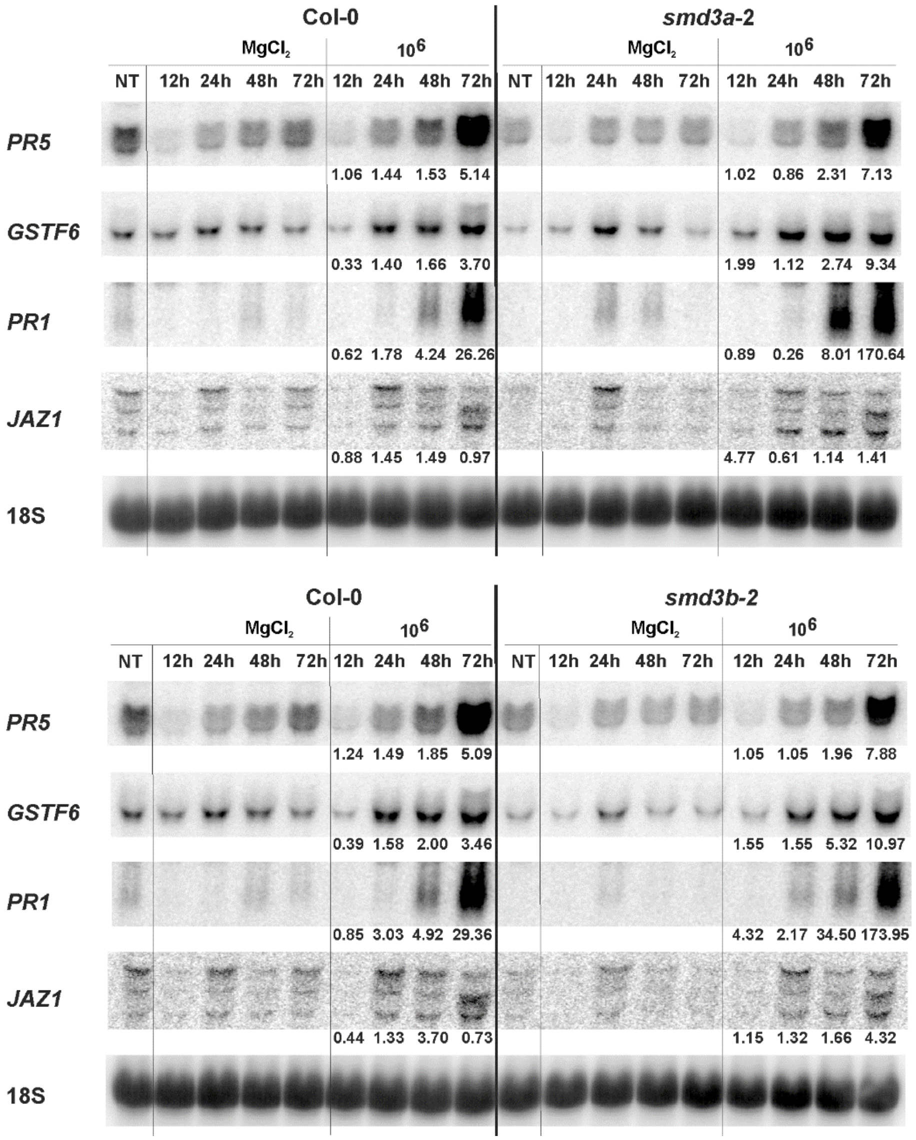

Supplementary Figure S3

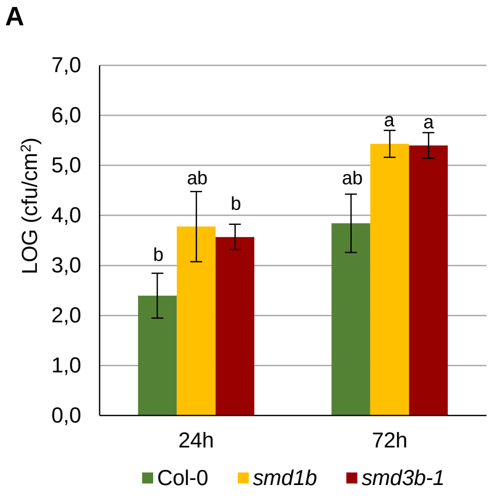

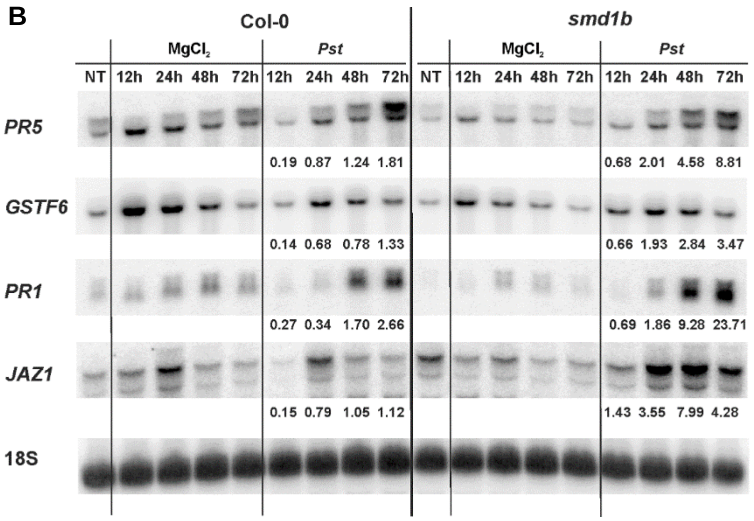

Supplementary Figure S4

A

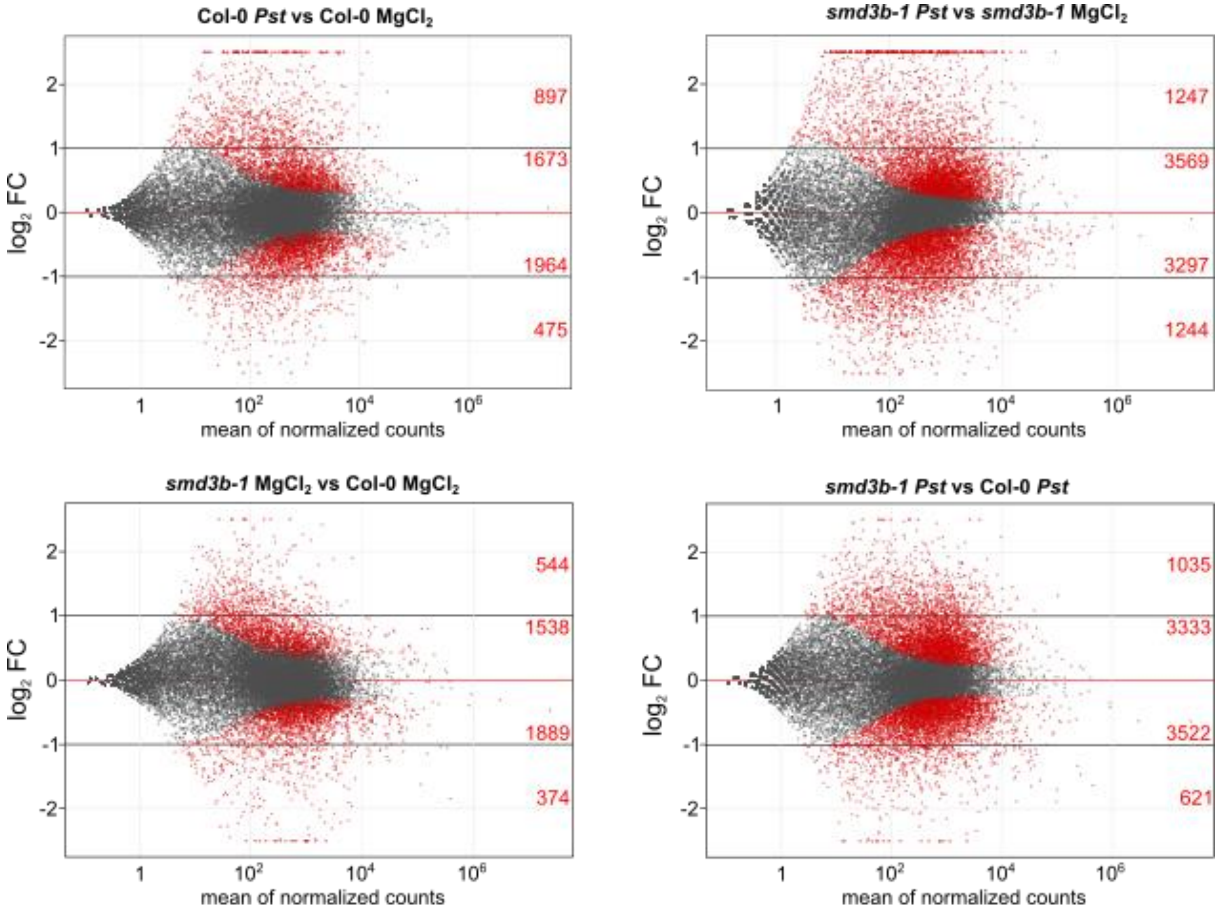

B

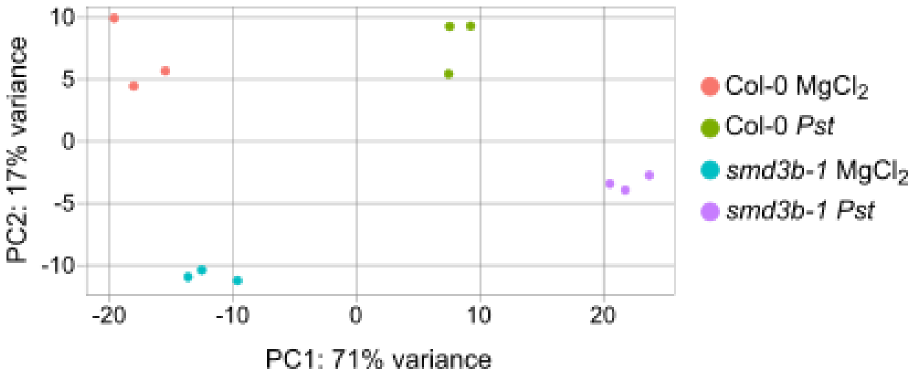

Supplementary Figure S5

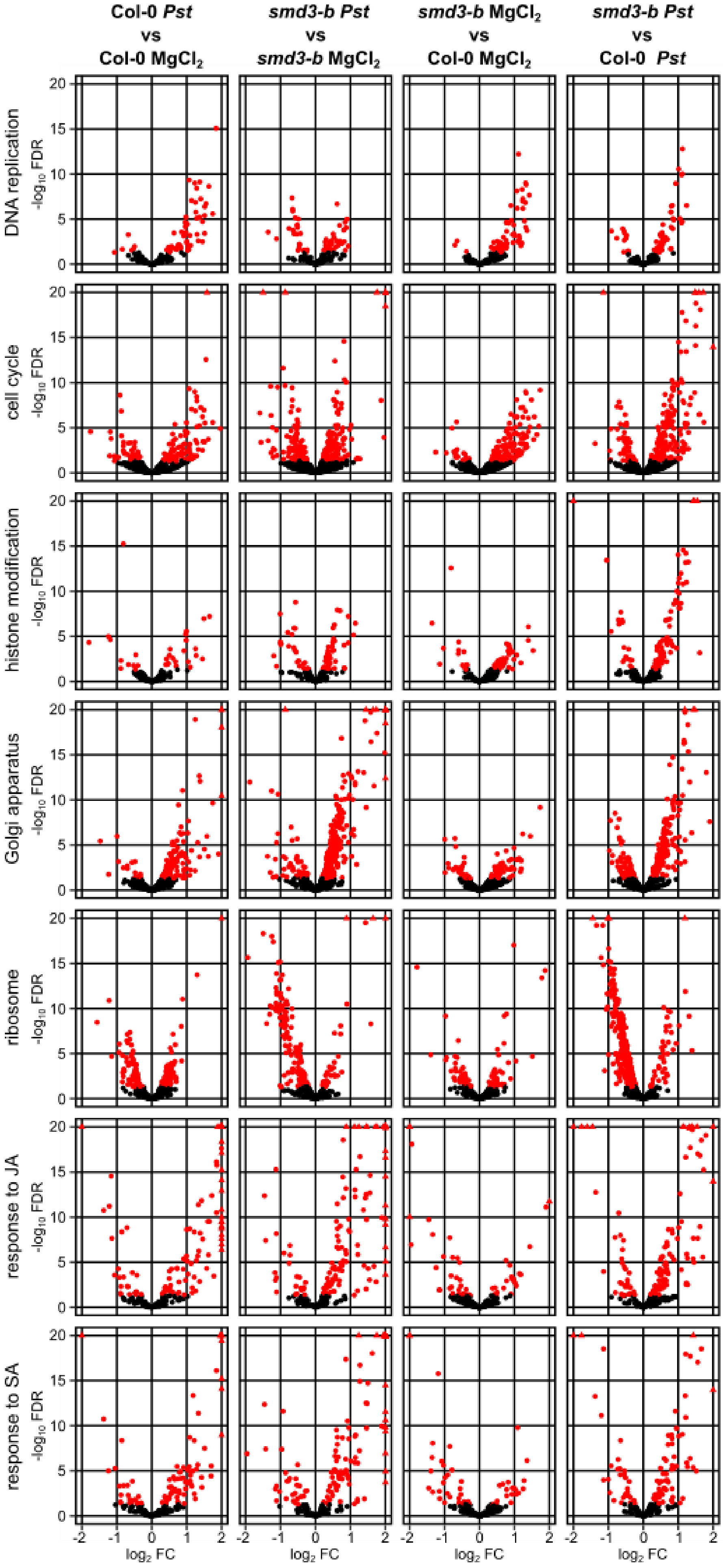

Supplementary Figure S6

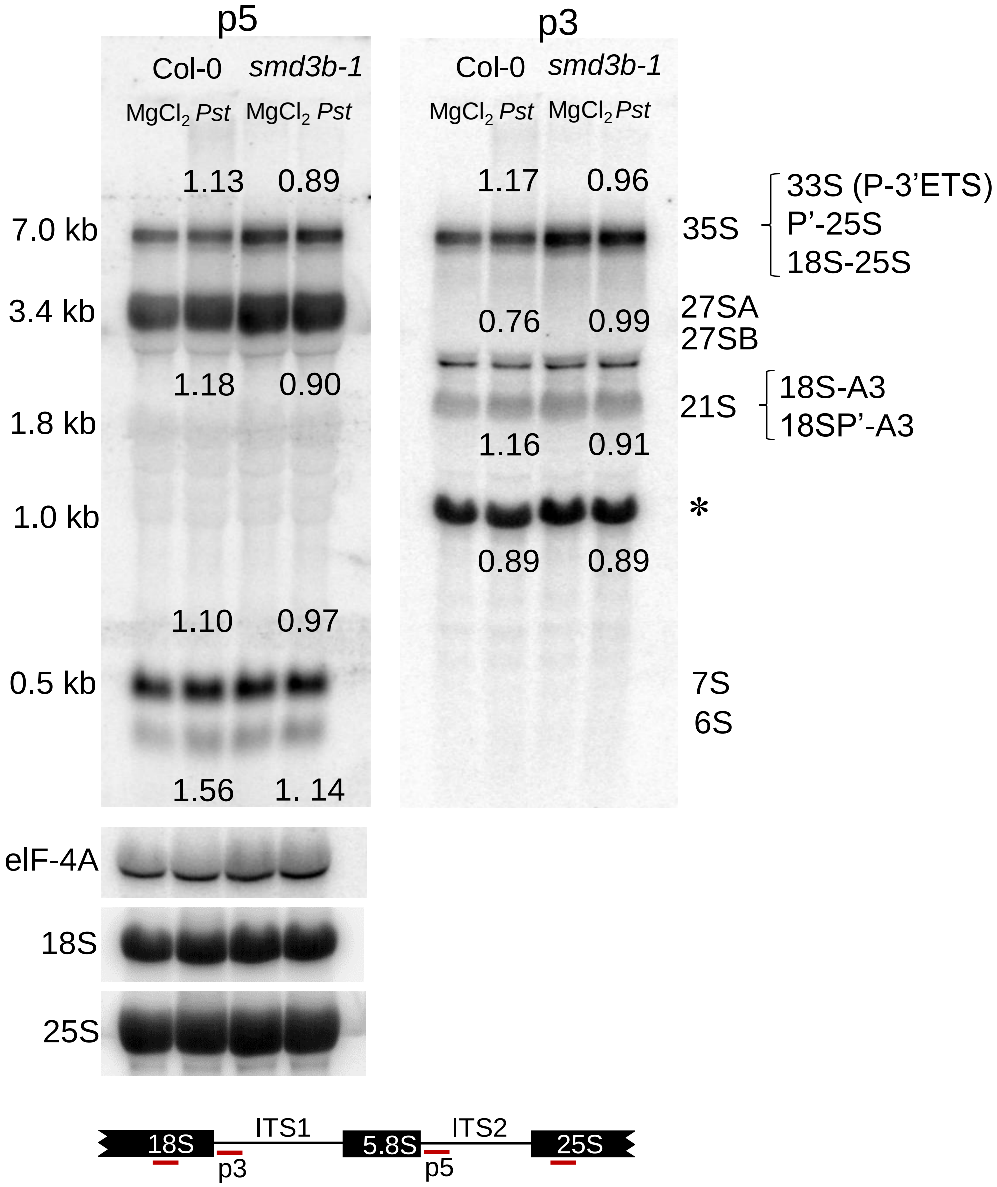

Supplementary Figure S7

A

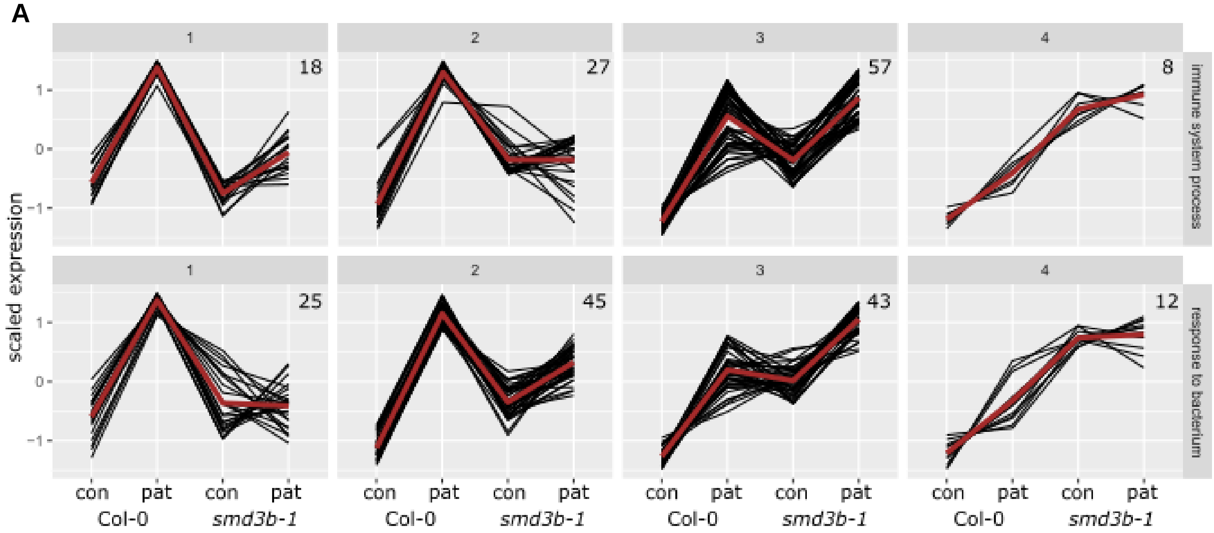

B

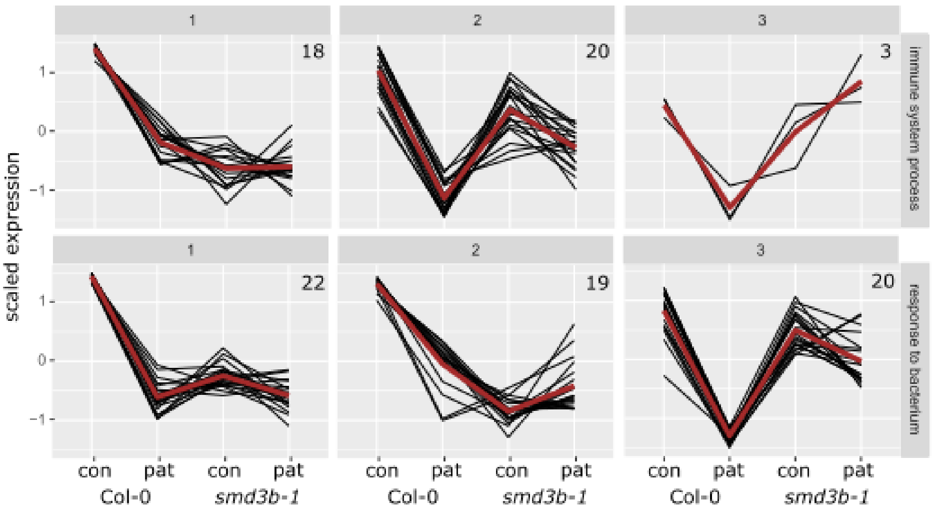

C

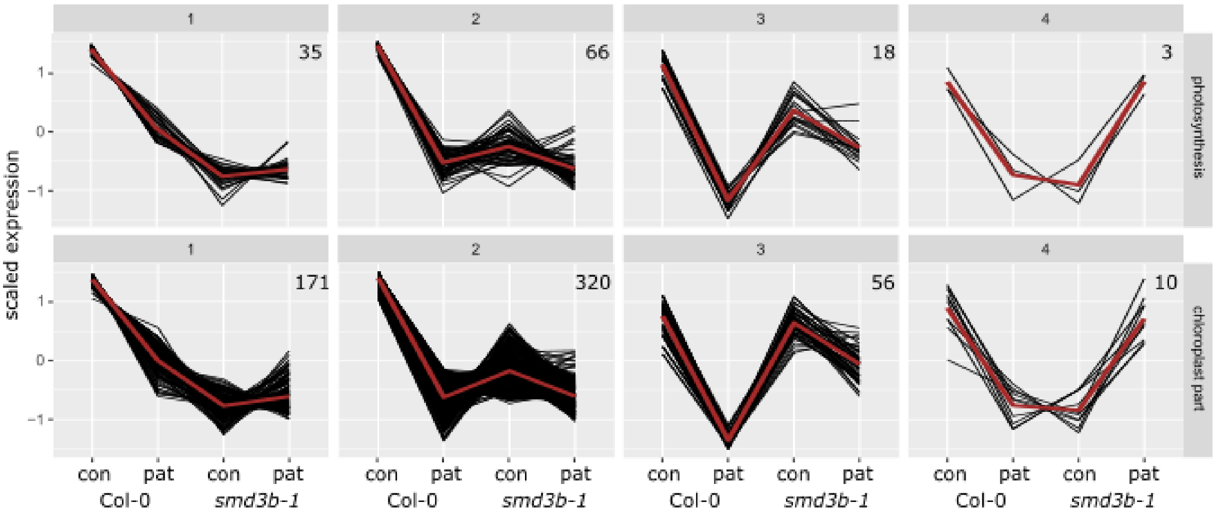

Supplementary Figure S8

A

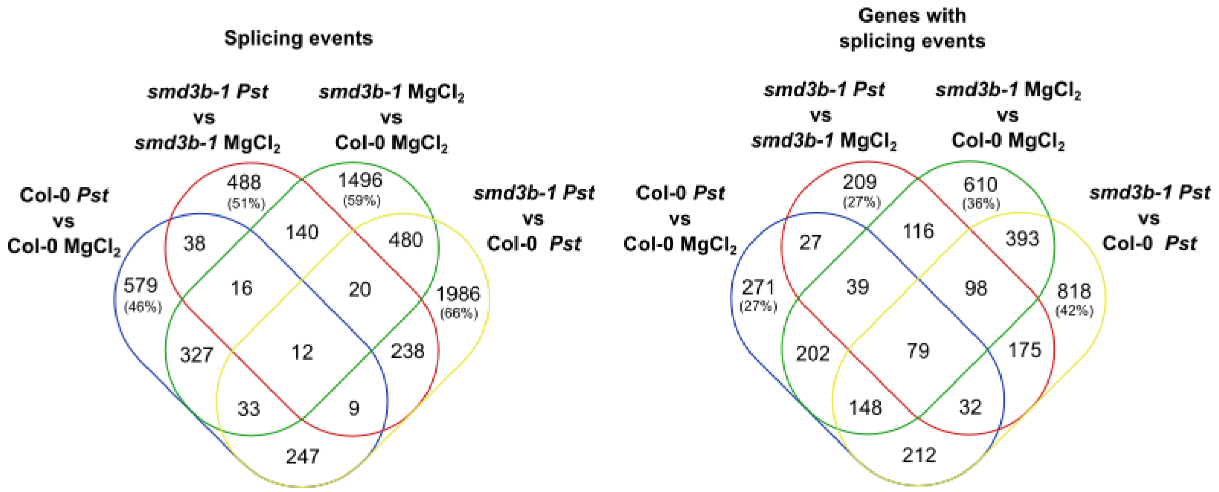

B

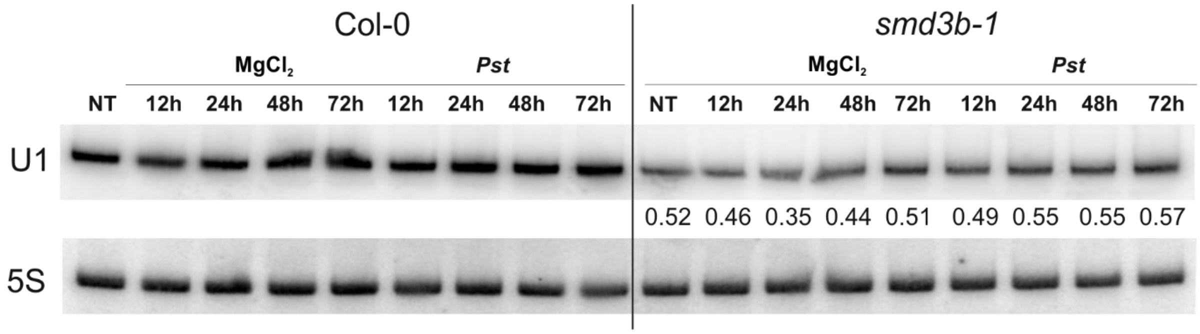

Supplementary Figure S9

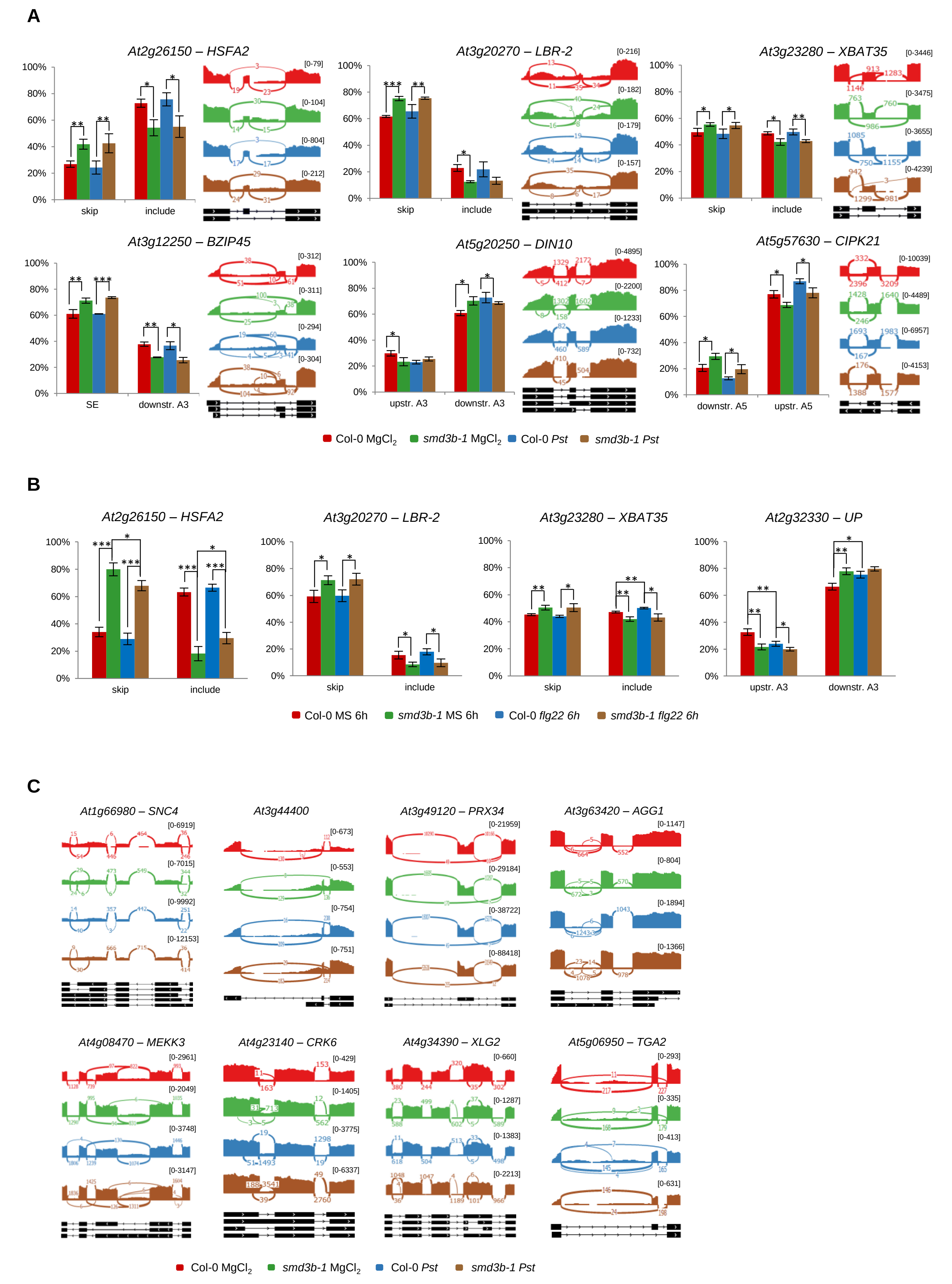

Supplementary Figure S10

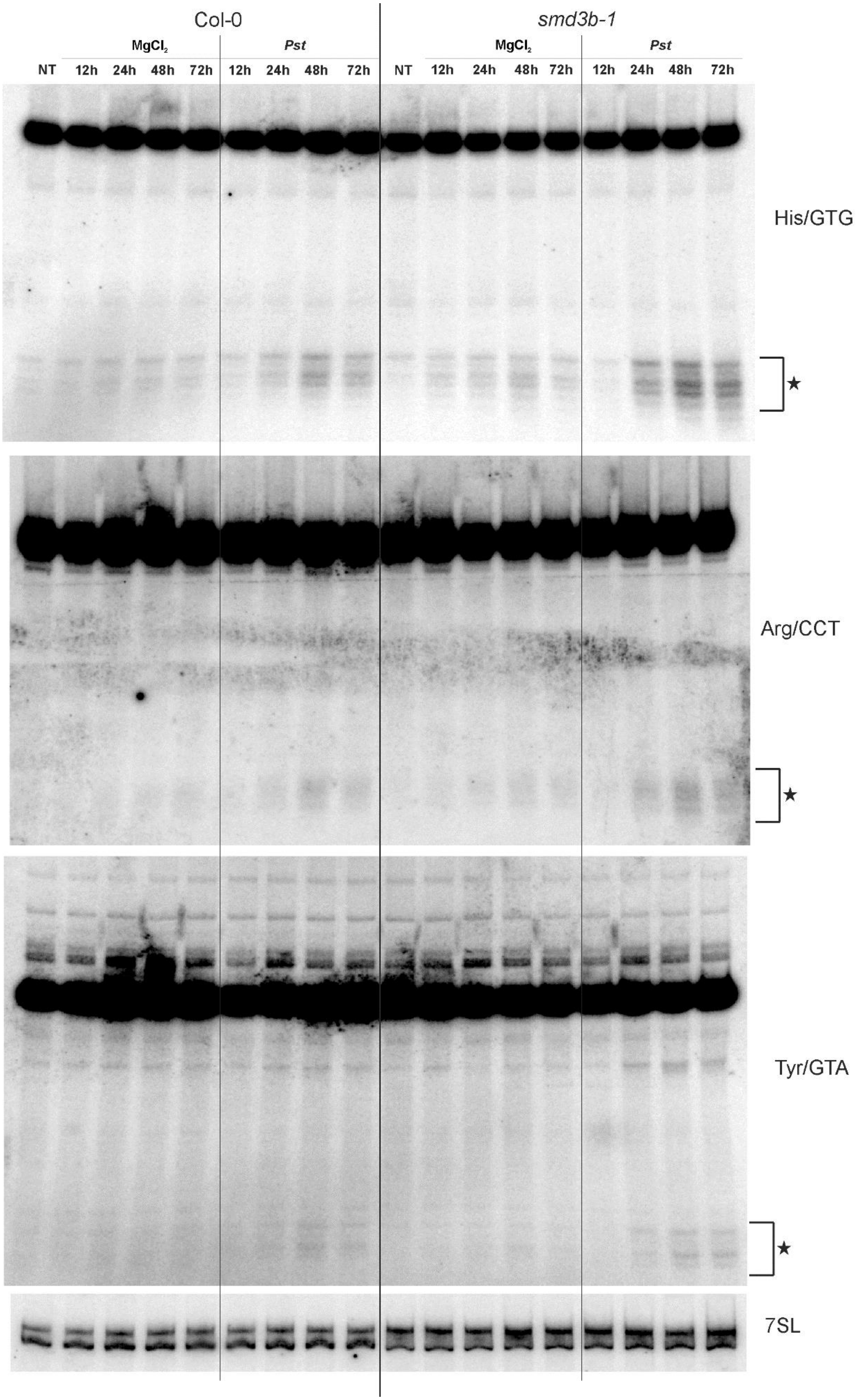

Supplementary Figure 11

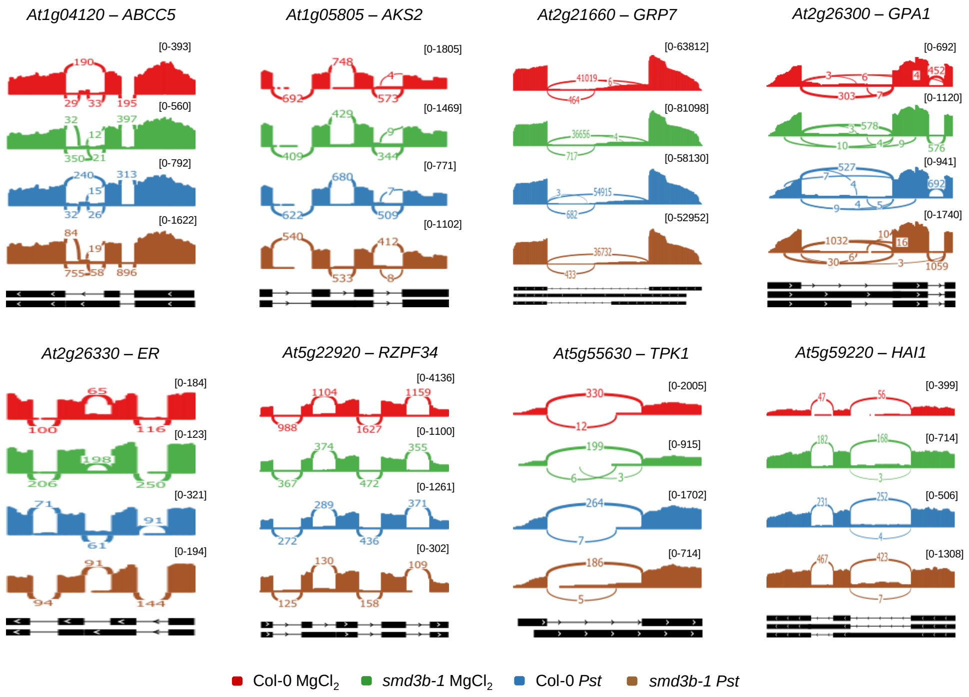

Supplementary Figure S12

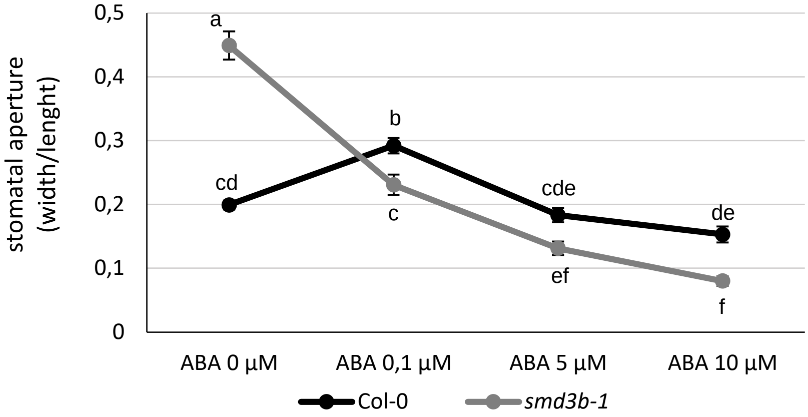

Supplementary Figure S13

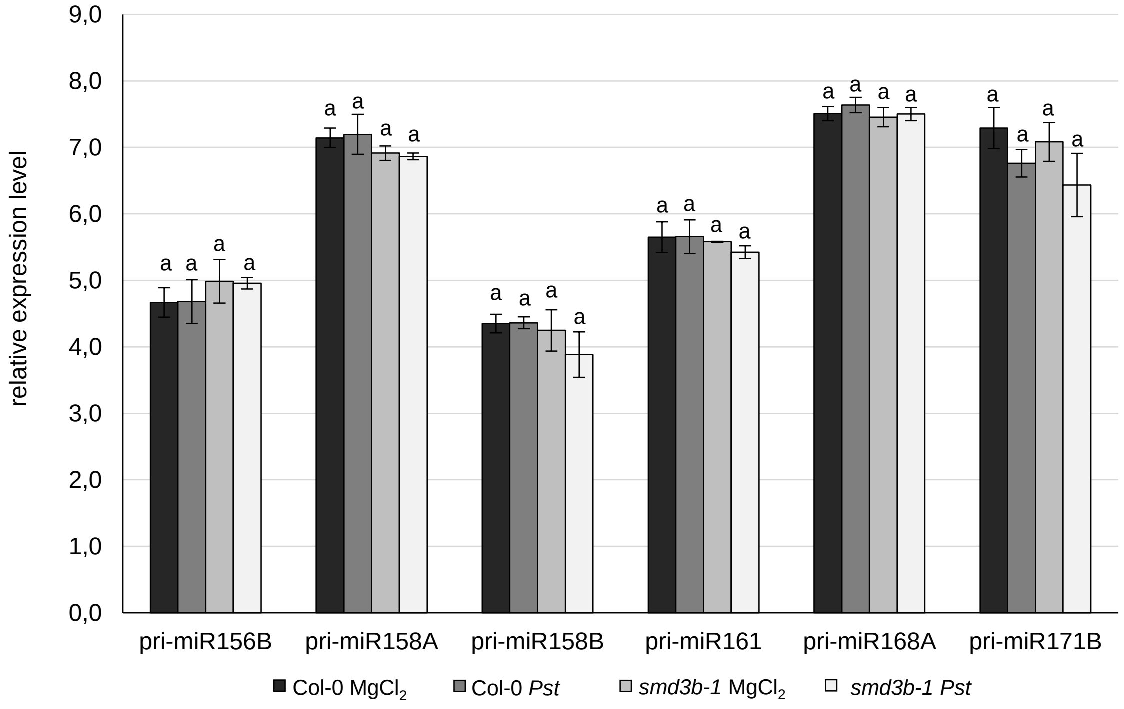

Supplement: Supplementary Figure 1 — The smd3b and smd3a mutations cause changes in response to infection. (A) Structure of the AtSMD3-a (At1g76300) and AtSMD3-B (At1g20580) genes. Exons are represented by gray bars, UTRs are illustrated by black bars and localization of T-DNA insertions are indicated. (B) Growth of Pst DC3000 after 24 and 72 hpi in Col-0, smd3a-2 and smd3b-2 mutants. For each time point leaf disks were collected from 5 plants. Results are mean of two independent experiments. (C) Disease symptoms in Col-0 and smd3b-1 6-week-old plants (72 hpi). Experiments were repeated at least four times; representative pictures are shown. (D) Northern blot analysis of factors involved in pathogen response (another biological replicate). Samples were collected from non-treated (NT), control (MgCl2) and infected (Pst) Col-0 and smd3b-1 plants at indicated time points. Numbers represent transcript level in Pst-treated Col-0 and the smd3b-1 relative to control and normalized to 18S rRNA loading control. (E) RT-qPCR analysis of selected genes involved in pathogen response. Mean values ± SEM were obtained from three independent experiments, letters represent significant difference (P < 0.05) for Tukey’s HSD test. UBC9 mRNA was used as a reference. [file Data_Sheet_1.zip › data sheet 1/Presentation 2.pdf]
