# Supplementary material for: Arabidopsis Spliceosome Factor SmD3 Modulates Immunity to Pseudomonas syringae Infection
Source: Front Plant Sci. 2021 Dec 3;12:765003. doi: 10.3389/fpls.2021.765003 (PMC8678131; doi:10.3389/fpls.2021.765003)
Supplement: Supplementary Figure 1 — The smd3b and smd3a mutations cause changes in response to infection. (A) Structure of the AtSMD3-a (At1g76300) and AtSMD3-B (At1g20580) genes. Exons are represented by gray bars, UTRs are illustrated by black bars and localization of T-DNA insertions are indicated. (B) Growth of Pst DC3000 after 24 and 72 hpi in Col-0, smd3a-2 and smd3b-2 mutants. For each time point leaf disks were collected from 5 plants. Results are mean of two independent experiments. (C) Disease symptoms in Col-0 and smd3b-1 6-week-old plants (72 hpi). Experiments were repeated at least four times; representative pictures are shown. (D) Northern blot analysis of factors involved in pathogen response (another biological replicate). Samples were collected from non-treated (NT), control (MgCl2) and infected (Pst) Col-0 and smd3b-1 plants at indicated time points. Numbers represent transcript level in Pst-treated Col-0 and the smd3b-1 relative to control and normalized to 18S rRNA loading control. (E) RT-qPCR analysis of selected genes involved in pathogen response. Mean values ± SEM were obtained from three independent experiments, letters represent significant difference (P < 0.05) for Tukey’s HSD test. UBC9 mRNA was used as a reference. [file Data_Sheet_1.zip › data sheet 1/Supplementary Table 1.PDF]

Table S1. Characteristics of genes involved in pathogen response having significantly affected alternative splicing events in the *smd3b-1* plants. Corresponds to Figs 3 and S9C, S11.

**ID**	**Name**	**Description**	**Reference**
At1g04120	*ABCC5* (*ATP-BINDING CASSETTE C5*)	encodes a high-affinity inositol hexakisphosphate transporter that plays a role in guard cell signaling and phytate storage. It is a member of MRP subfamily / ABC transporter subfamily C. Acts upstream of or within abscisic acid-activated signaling pathway involved in stomatal movement.	Nagy et al. (2009). The *Arabidopsis* ATP-binding cassette protein AtMRP5/AtABCC5 is a high affinity inositol hexakisphosphate transporter involved in guard cell signaling and phytate storage. *J. Biol. Chem.* 284:33614-22.
At1g05805	*AKS2* (*ABA-RESPONSIVE KINASE SUBSTRATE 2*)	ABA-RESPONSIVE KINASE SUBSTRATE 2, basic helix-loop-helix (bHLH) DNA-binding superfamily protein, regulation of stomatal movement	Takahashi et al. (2013) bHLH transcription factors that facilitate K^+ uptake during stomatal opening are repressed by abscisic acid through phosphorylation. *Sci. Signal.* 6:ra48
At1g53165	*MAP4K (MITOGEN-ACTIVATED PROTEIN KINASE KINASE KINASE KINASE4*)	In *Arabidopsis*, the MAP4K family contains 10 members. MAP4K family is involved in plant innate immunity.	Zhang et al. (2018). The MAP4 kinase SIK1 ensures robust extracellular ROS burst and antibacterial immunity in plants. *Cell Host Microbe* 24:379-391.
At1g66980	*SNC4 (SUPPRESSOR OF NPR1-1 CONSTITUTIVE 4)*	SNC4 leads to constitutive activation of defense responses. Analysis of suppressor mutants of *snc4-1D* identified two conserved splicing factors, SUA (SUPPRESSOR OF ABI3-5) and RSN2 (REQUIRED FOR SNC4-1D 2), that are required for the constitutive defense responses in *snc4-1D*. In *sua* and *rsn2* mutants, SNC4 splicing is altered and the amount of SNC4 transcripts is reduced. pre-mRNA splicing plays important roles in the regulation of plant immunity mediated by the RLKs SNC4 and CERK1.	Zhang et al. (2014). Splicing of receptor-like kinase-encoding SNC4 and CERK1 is regulated by two conserved splicing factors that are required for plant immunity. *Mol. Plant* 7:1766–1775.

At2g01450	*MPK17*<br>(*MAP KINASE 17*)	MAP kinase family member, function in control of peroxisome proliferation in salt stress.	Frick and Strader (2018). Kinase MPK17 and the peroxisome division factor PMD1 influence salt-induced peroxisome proliferation. *Plant Physiol.* 176:340-351.
At2g21660	*GRP7*<br>(*GLYCINE RICH PROTEIN 7*)	Encodes a small glycine-rich RNA binding protein that is part of a negative-feedback loop through which AtGRP7 regulates the circadian oscillations of its own transcript. GRP7 appears to promote stomatal opening and reduce tolerance under salt and dehydration stress conditions, but, promotes stomatal closing and thereby increases stress tolerance under conditions of cold tolerance. Loss of function mutations have increased susceptibility to pathogens suggesting a role in mediating innate immune response.	Kim et al. (2008). Glycine-rich RNA-binding protein 7 affects abiotic stress responses by regulating stomata opening and closing in *Arabidopsis thaliana*. *Plant J.* 55:455-466.
At2g25000	*WRKY60*	Pathogen-induced transcription factor involved in defense response to bacterium and fungus. Constitutive expression of WRKY18 enhanced resistance to *P. syringae*, its co-expression with WRKY40 or WRKY60 made plants more susceptible to both *P. syringae* and *B. cinereal.*	Xu et al. (2006). Physical and functional interactions between pathogen-induced *Arabidopsis* WRKY18, WRKY40, and WRKY60 transcription factors. *Plant Cell* 18: 1310-1326.
At2g26300	*GPA1*<br>(*G PROTEIN ALPHA SUBUNIT 1*)	Encodes an alpha subunit of a heterotrimeric GTP-binding protein. GPA1 is a positive regulator in ABA-mediated inhibition of stomatal opening.	Mishra et al. (2006). A bifurcating pathway directs abscisic acid effects on stomatal closure and opening in *Arabidopsis*. *Science* 321:264-266.
At2g26330	*ER*<br>*(ERECTA)*	ER has been identified as a quantitative trait locus for transpiration efficiency by influencing epidermal and mesophyll development, stomatal density and porosity of leaves. Together with ERL1 and ERL2, ER governs the initial	Lee et a. (2015). Competitive binding of antagonistic peptides fine-tunes stomatal patterning. *Nature* 522:439-443.

		decision of protodermal cells to either divide proliferatively to produce pavement cells or divide asymmetrically to generate stomatal complexes. ER binds to the peptides STOMAGEN and EPF2 which compete for the same binding site. The ER-EFP2 complex activates MAPK signaling that inhibits stomatal development.	
At3g44400		Disease resistance protein (TIR-NBS-LRR class) family	Katagiria et al. (2002). The *Arabidopsis thaliana-Pseudomonas syringae* interaction. *Arabidopsis* book. doi: 10.1199/tab.0039
At3g45860	*CRK4* (*CYSTEINE-RICH RLK 4*)	CRK4, CRK6, and CRK36 have been shown to interact with FLS2, and their overexpression leads to increased flg22 responsiveness or constitutive defenses including elevated ROS burst, defenses gene expression and disease resistance to *P. syringae.*	Yeh et al. (2015) Enhanced *Arabidopsis* pattern-triggered immunity by overexpression of cysteine-rich receptor-like kinases. Front. *Plant Sci*. 6:322.
At3g49120	*PRX34* (*PEROXIDASE 34*)	In *Arabidopsis*, class III apoplastic peroxidases PRX33 and PRX34 are essential for ROS production in response to flg22 and elf18.	Daudi et al. (2012) The apoplastic oxidative burst peroxidase in *Arabidopsis* is a major component of pattern-triggered immunity. *Plant Cell* 24:275-287.
At3g54230	*SUA* (*SUPPRESSOR OF ABI3-5*)	Encodes a splicing factor SUA. Analysis of suppressor mutants of *snc4-1D* identified two conserved splicing factors, SUA and RSN2 (REQUIRED FOR SNC4-1D 2), that are required for the constitutive defense responses in *snc4-1D*. In *sua* and *rsn2* mutants, SNC4 splicing is altered and the amount of SNC4 transcripts is reduced. SUA and RSN2 are also required for the proper splicing of CERK1 (CHITIN ELICITOR RECEPTOR KINASE1), which encodes another RLK that functions as a receptor for chitin.	Zhang et al. (2014). Splicing of receptor-like kinase-encoding SNC4 and CERK1 is regulated by two conserved splicing factors that are required for plant immunity. *Mol. Plant* 7:1766–1775.

		In *sua* and *rsn2* mutants, induction of reactive oxygen species by chitin is reduced and the non-pathogenic bacteria *Pseudomonas syringae* pv. *tomato* DC3000hrcC grows to higher titers than in wild-type plants.	
At3g63420	*AGG1* (*HETEROTRIMERIC G PROTEIN GAMMA-SUBUNIT*)	AGG1 and AGG2 positively regulate flg22-triggered apoplastic ROS production.	Gilroy et al. (2016) ROS, calcium, and electric signals: key mediators of rapid systemic signaling in plants. *Plant Physiol.* 171:1606-1615.
At4g08470	*MEKK3, MAPKKK10* (*MAPK/ERK KINASE KINASE* 3)	MEKK is another name for Mitogen-Activated Protein Kinase Kinase Kinase (MAPKKK or MAP3K). The *Arabidopsis* MEKK1-MKK1/MKK2-MPK4 kinase cascade is monitored by the nucleotide-binding leucine rich-repeat immune receptor SUMM2. Disruption of this kinase cascade leads to activation of SUMM2-mediated immune responses. MEKK1, MEKK2 and MEKK3 are located in a tandem repeat.	Nitta et al. (2020). MEKK2 inhibits activation of MAP kinases in *Arabidopsis*. *Plant J.* 103:705-714.
At4g16890	*SNC1* (*SUPPRESSOR OF NPR1-1, CONSTITUTIVE 1*)	Encodes a TIR-NB-LRR-type resistance gene involved in the salicylic acid-dependent defense response pathway. Mutant plants constitutively express pathogenesis-related (PR) genes and are pathogen resistant. Resistance signaling in *snc1* requires EDS1, MOS3 and PAD4.<br>Nuclear SNC1 functions in plant defense through its association with TPR1, a transcriptional corepressor. The activation of the R protein SNC1 leads to repression of miRNA and phasiRNA biogenesis to likely release the global repression of R gene expression to enable defense.	Cai et al. (2018). The disease resistance protein SNC1 represses the biogenesis of microRNAs and phased siRNAs. *Nat. Commun.* 9:5080.

At4g23140	*CRK6* (*CYSTEINE-RICH RLK* 6)	CRK6 and CRK7 are active kinases that are involved in signaling in response to extra-cellular ROS. CRK4, CRK6, and CRK36 have been shown to interact with FLS2, and their overexpression leads to increased flg22 responsiveness or constitutive defenses including elevated ROS burst, defenses gene expression and disease resistance to *P. syringae.*	Idanheimo et al. (2014). The *Arabidopsis thaliana* cysteine-rich receptor-like kinases CRK6 and CRK7 protect against apoplastic oxidative stress. *Biochem. Biophys. Res. Commun*. 445:457-462.
At4g25230	*RIN2* *(RPM1 INTERACTING PROTEIN 2)*	RPM1 interacting protein 2, has a CUE domain which is sufficient for the interaction with RPM1. Positive regulator of RPM1 and PRS2 mediated hypersensitive response. Functions as ubiquitin ligase and binds to RPM1.	Kawasaki et al. (2005). A duplicated pair of *Arabidopsis* RING-finger E3 ligases contribute to the RPM1- and RPS2-mediated hypersensitive response. *Plant J.* 44:258-270
At4g28910	*NINJA* *(NOVEL INTERACTOR OF JAZ)*	Involved in jasmonic acid mediated signaling pathway. NINJA forms a repressor complex by binding to JAZ and MYC, thereby inhibiting JA responses in the absence of ligands.	Pauwels et al. (2010). NINJA connects the co- repressor TOPLESS to jasmonate signalling. *Nature*, 464:788–791.
At4g33430	*BAK1* *(BRI1-ASSOCIATED RECEPTOR KINASE)*	BAK1 a leucine- rich repeat receptor-like kinase regulate the brassinosteroid receptor BRI1, is involved in signalling by FLS2 and EFR. FLS2 and BAK1 form a complex in vivo, in a specific ligand-dependent manner, within the first minutes of stimulation with flagellin.	Chinchilla et al. (2007). A flagellin-induced complex of the receptor FLS2 and BAK1 initiates plant defence. *Nature* 448:497-500.
At4g34390	*XLG2* (*EXTRA-LARGE G PROTEIN 3*)	XLG2 associates with FLS2 to stabilize BIK1, and XLG2 phosphorylation by BIK1 promotes ROS production, likely via XLG2-RBOHD association.	Liang et al. (2016) *Arabidopsis* heterotrimeric G proteins regulate immunity by directly coupling to the FLS2 receptor. *Elife* 5:e13568.
At5g06950	*TGA2* (*TGACG SEQUENCE-*	Transcription factor of the B-ZIP family that has high affinity for C-box motifs. Interacts with	Zhang et al. (2003). Knockout analysis of *Arabidopsis*

	SPECIFIC BINDING PROTEIN 2)	NPR1 and may regulate PR gene expression. Phosphorylated by a CK2-like protein in vitro. Phosphorylation is enhanced by salicylic acid treatment.	transcription factors TGA2, TGA5, and TGA6 reveals their redundant and essential roles in systemic acquired resistance. *Plant Cell* 15:2647–2653.
At5g22920	*CHYR1*/*RZPF34* (*CHY ZINC-FINGER AND RING PROTEIN 1/RING ZINC-FINGER PROTEIN 34*)	Encodes a protein with sequence similarity to RING, zinc finger proteins. Loss of function mutations show reduced (15%) stomatal aperture under non stress conditions.	Ding et al. (2015). Arabidopsis RZFP34/CHYR1, a ubiquitin E3 ligase, regulates stomatal movement and drought tolerance via SnRK2.6-mediated phosphorylation. *Plant Cell* 27:3228-3244.
At5g55630	*TPK1* (*TWO PORE K CHANNEL 1*)	Encodes AtTPK1 (KCO1), a member of the *Arabidopsis* thaliana K+ channel family of AtTPK/KCO proteins. Acts upstream of or within regulation of stomatal movement.	Isner et al. (2018). KIN7 kinase regulates the vacuolar TPK1 K + channel during stomatal closure. *Curr. Biol* 28:466-472.
At5g66210	*CPK28* (*CALCIUM-DEPENDENT PROTEIN KINASE 28*)	CPK28 specifically phosphorylates conserved residues in PUB25/26 to enhance their activity and promote BIK1 degradation. Further CPK28 destabilizes BIK1 following flg22 treatment. Flg22 treatment additionally induces dissociation of the G proteins from the FLS2-BIK1 complex and increased phosphorylation of PUB25/26 by CPK28.	Wang et al. (2018). A regulatory module controlling homeostasis of a plant immune kinase. *Mol. Cell* 69:493–504.
At5g59220	*HAI1 (HIGHLY ABA-INDUCED PP2C GENE 1)*	Encodes a member of the PP2C family (Clade A protein phosphatases type 2C). Functions as a negative regulator of osmotic stress and ABA signaling. Acts upstream of or within stomatal movement.	Antoni et al. (2012). Selective inhibition of Clade A phosphatases type 2C by PYR/PYL/RCAR abscisic acid receptors. *Plant Physiol.* 158:970-980.
