# Supplementary material for: Arabidopsis Spliceosome Factor SmD3 Modulates Immunity to Pseudomonas syringae Infection
Source: Front Plant Sci. 2021 Dec 3;12:765003. doi: 10.3389/fpls.2021.765003 (PMC8678131; doi:10.3389/fpls.2021.765003)
Supplement: Supplementary Figure 1 — The smd3b and smd3a mutations cause changes in response to infection. (A) Structure of the AtSMD3-a (At1g76300) and AtSMD3-B (At1g20580) genes. Exons are represented by gray bars, UTRs are illustrated by black bars and localization of T-DNA insertions are indicated. (B) Growth of Pst DC3000 after 24 and 72 hpi in Col-0, smd3a-2 and smd3b-2 mutants. For each time point leaf disks were collected from 5 plants. Results are mean of two independent experiments. (C) Disease symptoms in Col-0 and smd3b-1 6-week-old plants (72 hpi). Experiments were repeated at least four times; representative pictures are shown. (D) Northern blot analysis of factors involved in pathogen response (another biological replicate). Samples were collected from non-treated (NT), control (MgCl2) and infected (Pst) Col-0 and smd3b-1 plants at indicated time points. Numbers represent transcript level in Pst-treated Col-0 and the smd3b-1 relative to control and normalized to 18S rRNA loading control. (E) RT-qPCR analysis of selected genes involved in pathogen response. Mean values ± SEM were obtained from three independent experiments, letters represent significant difference (P < 0.05) for Tukey’s HSD test. UBC9 mRNA was used as a reference. [file Data_Sheet_1.zip › data sheet 1/Supplementary Table 2.PDF]

Northern blot

Name	Gene ID	Primer Forward	Primer Reverse
BAK1	At4g33430	AGTGGAGCAGCTAATCCAAGTGGC	GGTAAGAAAAGAAACCTGACGCACGG
BGL2	At3g57260	CAATGCAGAACATCGAGAACGCGG	CACCACGATTTCCAACGATCCGCC
CORI3	At4g23600	GCGAACTGGTTGGCTCACGC	TGGAATGGCGATCGCAGAAGC
FRK1	At2g19190	TCGGATTCGGCGTTTGTTGATTC	CTCTCGTTTCGCGCTGTTTCTGC
GSTF6	At1g02930	CTCAACTGGCAAGGACATGGCG	CATTCAAATCAAACACTCGGCAGCAG
JAZ1	At1g19180	ACGTCAGCCGACAACAACCATGAG	AGGGTTTGAAGACGCTTTGGCTGG
JAZ3	At3g17860	GCAGTTTCCATGTCGATGCCGGG	CGCTTTTGTGGCTCCGAGTCCG
MYC2	At1g32640	TGGACCACCGACGACAACGC	CCGACGGAGCAACACCACCG
PDF1.2	At5g44420	TGGCTAAGTTTGCTTCCATCATCACCC	TGGGACGTAACAGATACACTTGTGTGC
PR1	At2g14610	TCCCTCGAAAGCTCAAGATAGCCCAC	GCTTCTCGTTCACATAATTCCCACGAG
PR2	At3g57260	CAATGCAGAACATCGAGAACGCGG	CACCACGATTTCCAACGATCCGCC
PR4	At3g04720	TTCTCCGACCAACAACTGTC	ATATCAAACGCGATCAATGG
PR5	At1g75040	CGCCGGTCAAGGACCCAAGC	ACAGGCACTCTTGCAGGCCAC
VSP2	At5g24770	GACTTCGACACGGTGCCCGC	GGTCACGCCAGCAGCTTCGAG
WRKY29	At4g23550	AGGAGTTATTACAGATGCAG	TACCCACTGAAGAACTCTTG
elF-4A	At3g13920	TCATGAGAGCTTTGATGCCATGG	GATGAGAACACGGGAGGAACCAG

miRNA163		ATCGAAGTTCCAAGTCCTCTTCAA	
miRNA319		AGGGAGCTCCCTTCAGTCCAA	
miRNA393		GGATCAATGCGATCCCTTTGGA	
tRNA His/GTG		AACGTGGAATTCTAACCACTAAACTACAGCCAC	
tRNA Arg/CCT		AGGAAACAGACGCTCTATCCACTGAGCTACAGGCGC	
tRNA Tyr/GTA		TACAGTCTTCCGCTCTACCA	
p3		GGTCGTTCTGTTTTGGACAGGTATC	
p5		GCAAAGGATGGTGAGGGACGACG	
5S		GCACGCTTAACTGCGGAGTTCTG	
7SL		ACTGGGCAGCCCAGAAACATGC	
18S		GATCCTTCCGCAGGTTCACCTACG	
25S		CTCCGCTTATTGATATGCTTAAAC	
U1		GACCCCGTCCAGGTAAGTAAT	

RT-qPCR

Name	Gene ID	Primer Forward	Primer Reverse
EDS5	At4g39030	GGATCCCGGTTAACTCCCACC	TGATCGCACCGAGTGTTCTCC
FAMT	At3g44860	AGGTCCCAACAGTTACCGGG	CCAGGTGGGTCTTCTTGCATC
FLS2	At5g46330	TGCAAGCCTTAAGTCGCTTTCG	AACAGCTCTCCAGGGATGGTTC
GAPDH	At1g13440	TGGAAAATTGACCGGAATGT	TCGTCGTATGTTGCAGCTTT

GSTF6	At1g02930	TGGTCGATCTTCACACTATCCCTGTG	GATGTCAGCAACCCAAGCACTCA
HEL	At3g04720	GTACGCGCCACCTACCATTTC	GGAGCAATAAGCACTCACGGC
JAZ1	At1g19180	GCGATCCAGCCAAAGCGTCT	CTTGGTACGGCTTGAGGGTGGT
JAZ9	At1g70700	AGAAGACGTTAGGGCGTCTCTG	CTTGACAGACATGAGTCCATGACA
NPR1	At1g64280	GGAGTATCCGTTAGACCCCGAG	GGTTTTCTCCATCCCATCTTGCC
NPR3	At5g45110	TGCTGCGATGCGTAGAGAGC	TGCGCTACGTCCGTCAGATG
PAD4	At3g52430	TGCCTGAGGAGTGTGTGAGAAG	AAGTATCTTGCGTTGTGCTCGC
PR1	At2g14610	CAGGCACGAGGAGCGGTAGG	CCCCGTAAGGCCCACCAGAG
PR5	At1g75040	GCCTGCAAGAGTGCCTGTGAG	CGAGTAGTCCGTGGGAGGACAAG
RIN4	At3g25070	TCTCCCATGGCACGTTCG	AGGAACATTCTCCTCAGCTTCCC
SGT1	At2g43820	GGCTCGACACAAGGCCACA	CTGACCACCCACAGGAAGCTG
SID2	At1g74710	TTGGCAGGGAGACTTACGAAGG	CTTGCTTCTTCTGCTGGAAGCC
UBC9	At4g27960	TTCATGTAGCGCAGGACCCGTTG	ACTCCTCCAGAATAAGGGCTATCCG
WKRY70	At3g56400	CCAACGCAGAAACTCCCAAGAGC	CTCCGTGGACGAACCATGATGAC
WRKY46	At2g46400	CTGCACCTGCTGCTGTTGAGA	CGACCACAACCAATCCTGTCC
WRKY53	At4g23810	GAGATCAGACGGGGATGCTACGG	CGGCGAGGCTAATGGTGGTGT
	At2g26150	GGAAATGGAGGAAGAAACGG	GCCTCAACCTAACTACCTCAG
	At3g20270	CTCAGGGATTACTTACTACC	CTCCGCGGAATAAATTAGCCC
	At3g23280	GCACAGTGATGCCTTTGTGG	GGCTGTCACCTTCAGTCGAAGG
	At3g12250	GGACAACCTTTTACTCAGACAGG	GGTATCAGCCATACTAGTTTCTG
	At5g20250	CCATTCAAATCTCACATTCCC	CCCGTCGGAAATACGAACCGCC
	At5g57630	CAGATGCTAGAAAACTTTTCC	CCATACATCTACTGCTGCTCC
pri-miR156A		GAAAGAGTTGGGACAAGAGAAACG	AGAGAACGAAGACAGGCCAAAGA
pri-miR156B		GGGAGAGATGGTGATTGAGGAATGC	GTGAGCACGCACACGCAAAGT
pri-miR158A		TGAACAAGGGCATCTAAAGTCACA	TTACTGGACCACGAATTCACCAT
pri-miR158B		GACTGCAGAAGGATAAGAACACG	ACATTTGGGGAAAAGCAATG
pri-miR161		TGACCAGTTTATTGCGTCGATCA	TGCTTTTCCCTCTTTTTACAAATGC
pri-miR163		AGTACCTTAGATAAACCGACCAAAACC	AACCGGGAACTCCAGCACTT
pri-miR168A		ATTCGCTTGGTGCAGGTC	TCCGATTCAGTTGATGCAAG
pri-miR171A		TGGCCTGGTTCACTCAGATCTTA	ACAAGACCACAAAGTCCAAAATAGAGA
pri-miR171B		TTTGTTTATTGGTTTTCTGGAGCTAAG	CAACACCGTTCTCCATGAGTTAAGA
pri-miR171C		ATGTGGATGGAGTTTGGTGTAA	GTGATATTGGCACGGCTCA
pri-miR173		GGTGATTAAGTACTTTCGCTTGC	GCAAGCTCTTTCGCTTACACA
pri-miR393A		GCTTGGTTTTGGATCATGCTATCTCTT	CGGAATCTTAAAGCCAGCAAAG
pri-miR393B		AGGGATCGCATTGATCCTAA	CGCATGATCCGGAAAAGTAA
pri-miR403		AGAGTCGTATTACATGTTTTGTGCTTGA	ACAGATTACGAGTTTGTGCGTGAA
